# A Novel Application of Mixed Effects Models for Reconciling Base-Pair Resolution 5-Methylcytosine and 5-Hydroxymethylcytosine Data in Neuroepigenetics

**DOI:** 10.3389/fgene.2019.00801

**Published:** 2019-09-10

**Authors:** Joseph Kochmanski, Candace Savonen, Alison I. Bernstein

**Affiliations:** Department of Translational Neuroscience, College of Human Medicine, Michigan State University, Grand Rapids, MI, United States

**Keywords:** neuroepigenetics, mixed effects model for repeated measures, 5-methylcytosine, 5-hydroxymethylcytosine, bioinformatics

## Abstract

Epigenetic marks operate at multiple chromosomal levels to regulate gene expression, from direct covalent modification of DNA to three-dimensional chromosomal structure. Research has shown that 5-methylcytosine (5-mC) and its oxidized form, 5-hydroxymethylcytosine (5-hmC), are stable epigenetic marks with distinct genomic distributions and separate regulatory functions. In addition, recent data indicate that 5-hmC plays a critical regulatory role in the mammalian brain, emphasizing the importance of considering this alternative DNA modification in the context of neuroepigenetics. Traditional bisulfite (BS) treatment-based methods to measure the methylome are not able to distinguish between 5-mC and 5-hmC, meaning much of the existing literature does not differentiate these two DNA modifications. Recently developed methods, including Tet-assisted bisulfite treatment and oxidative bisulfite treatment, allow for differentiation of 5-hmC and/or 5-mC levels at base-pair resolution when combined with next-generation sequencing or methylation arrays. Despite these technological advances, there remains a lack of clarity regarding the appropriate statistical methods for integration of 5-mC and 5-hmC data. As a result, it can be difficult to determine the effects of an experimental treatment on 5-mC and 5-hmC dynamics. Here, we propose a statistical approach involving mixed effects to simultaneously model paired 5-mC and 5-hmC data as repeated measures. We tested this approach using publicly available BS/oxidative bisulfite-450K array data and showed that our new approach detected far more CpG probes with paired changes in 5-mC and 5-hmC by Alzheimer’s disease status (n = 14,183 probes) compared with the overlapping differential probes generated from separate models for each epigenetic mark (n = 68). Of note, all 68 of the overlapping probe IDs from the separate models were also significant in our new modeling approach, supporting the sensitivity of our new analysis method. Using the proposed approach, it will be possible to determine the effects of an experimental treatment on both 5-mC and 5-hmC at the base-pair level.

## Introduction

### Epigenetics

Epigenetic marks operate at four major levels—DNA modifications, histone modifications, noncoding RNAs, and three-dimensional chromatin structure (Chen et al., 2017b). The most studied DNA modification is 5-methylcytosine (5-mC), the addition of a methyl group at the C5 position of a cytosine in the DNA sequence (Moore et al., 2013). An abundance of research shows associations between 5-mC and gene expression and suggests that this epigenetic mark plays a key role in transcriptional control (Moore et al., 2013). In addition to 5-mC, there are three further oxidized DNA modifications—5-hydroxymethylcytosine (5-hmC), 5-formylcytosine (5-fC), and 5-carboxylcytosine (5-caC) (Shen et al., 2014). These alternative DNA modifications are formed when 5-mC is successively oxidized by the ten-eleven translocase (Tet) family of proteins (Shen et al., 2014). The 5-fC and 5-caC modifications are rapidly removed by thymine-DNA glycosylase and base excision repair and are thought to be transient (He et al., 2011; Ito et al., 2011; Maiti and Drohat, 2011). In contrast, 5-hmC can be a stable epigenetic mark that regulates transcription (Hahn et al., 2014). In particular, 5-hmC appears to play an important role in the central nervous system, where it is present at much higher levels than embryonic stem cells and other somatic tissues (Globisch et al., 2010; Szwagierczak et al., 2010; Nestor et al., 2012; Cheng et al., 2015).

### Neuroepigenetics: A Unique Role for 5-Hydroxymethylcytosine

Given the relative enrichment of 5-hmC in nervous tissue, an abundance of new research has examined the potential regulatory role of 5-hmC in the brain. Studies show that 5-hmC is acquired during neuronal development (Hahn et al., 2013; Szulwach et al., 2011) and maintained throughout adulthood (Chen et al., 2014). In the brain, 5-hmC has a specific distribution across the genome, with enrichment at genic regions, distal regulatory elements, and exon–intron boundaries (Khare et al., 2012; Lister et al., 2013; Wen et al., 2014). At the level of specific genes, 5-hmC is enriched in gene bodies of genes that are transcriptionally active in neuronal tissue (Mellén et al., 2012). In addition, different anatomical regions of the brain show distinct 5-hmC patterning (Lunnon et al., 2016), suggesting a specific regulatory role for this epigenetic mark.

Recent work also highlights that 5-mC and 5-hmC differ in their genomic distribution in the nervous system (Chen et al., 2014; Cheng et al., 2015). During synaptogenesis, 5-hmC preferentially accumulates in euchromatin, whereas 5-mC gradually builds up in heterochromatic regions (Chen et al., 2014). In addition, 5-mC and 5-hmC preferentially recruit distinct sets of DNA-binding proteins in brain tissue (Spruijt et al., 2013). For example, whereas Mbd1, Mbd4, and MeCP2 bind 5-mC at higher affinity, Neil1, Thy28, and Wdr76 have a higher affinity for 5-hmC (Spruijt et al., 2013). 5-hmC is also preferentially bound by the DNA-binding protein Uhrf2 in neuronal progenitor cells (Spruijt et al., 2013), a process that may regulate spatial memory and learning (Chen et al., 2017a). The distinct sets of readers for 5-hmC and 5-mC indicate that these two epigenetic marks have separate regulatory functions in neuronal tissue.

Combined, the available data suggest that 5-hmC plays a critical regulatory role in the mammalian brain, emphasizing the importance of considering this alternative DNA modification in the context of neuroepigenetics. As such, it is critical that the field develops methods to accurately distinguish 5-hmC from 5-mC in a genome-wide context. Here, we discuss the available methods for measuring 5-hmC, including their strengths and weaknesses, and then propose a statistical approach for co-analyzing the effects of an experimental treatment on paired 5-mC and 5-hmC data.

### Differentiation of Base-Pair Resolution 5-Methylcytosine and 5-Hydroxymethylcytosine

**Figure 1:**
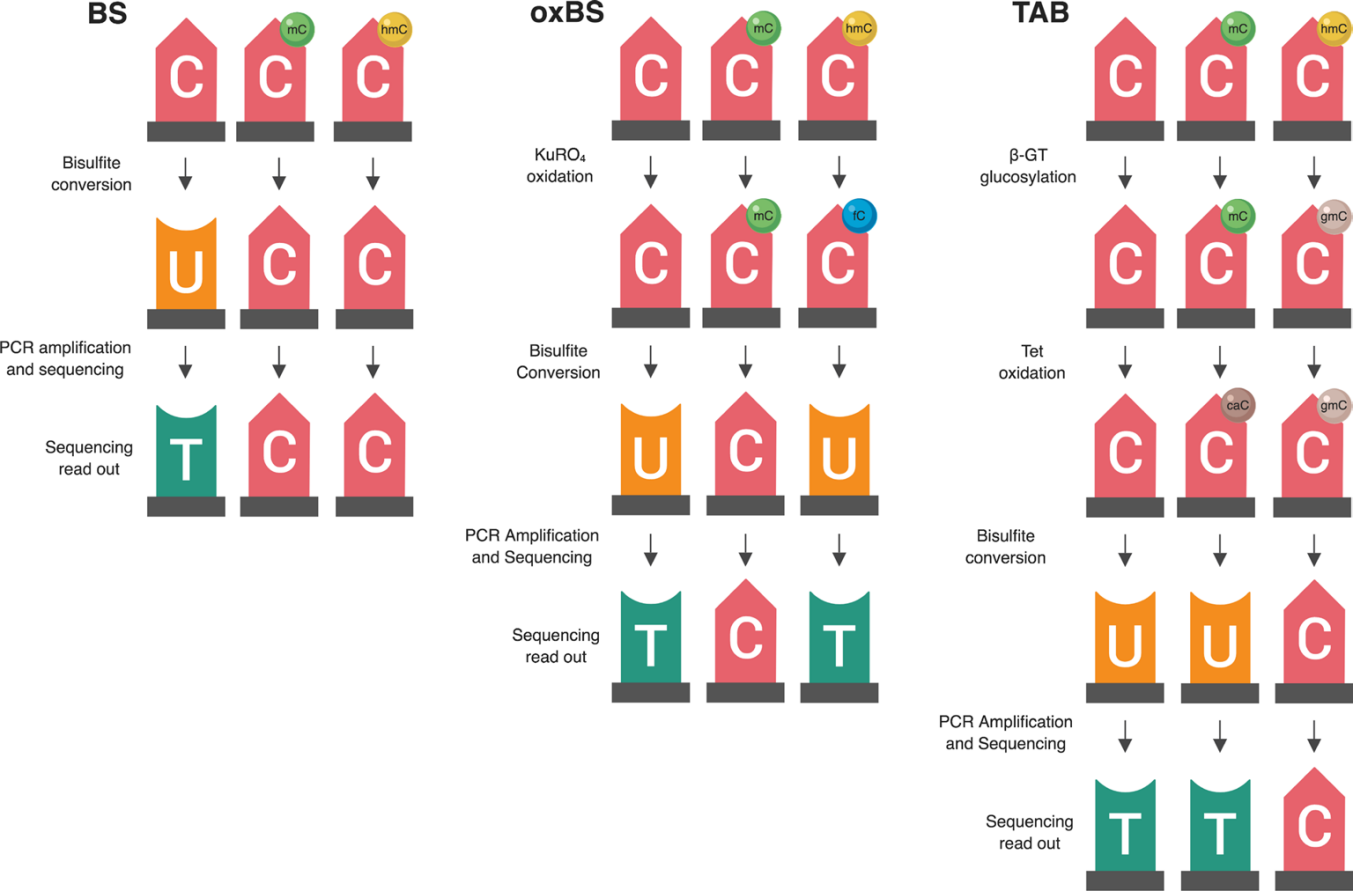
Summary of two available methods for measuring genomic 5-hmC levels. There are two widely adopted methods used to measure 5-hmC levels at the base-pair level—paired BS/oxBS and TAB (Yu et al., 2012; Booth et al., 2013). These two methods differ in their chemistry and data interpretation. In the oxBS method, KRuO_4_ oxidation selectively converts 5-hmC to 5-fC, which is removed during BS conversion. By comparing oxBS data (5-mC) with traditional BS data, it is possible to infer 5-hmC levels. For the TAB treatment method, 5-hmC is selectively tagged with a β-glucosyl group, which makes it resistant to either BS conversion. On its own, TAB provides a true value for 5-hmC but does not measure 5-mC.

Historically, the majority of neuroepigenetics studies investigating DNA modifications utilized bisulfite (BS) treatment-based methods to measure DNA methylation (Rein et al., 1998; Clark et al., 2006; Beck and Rakyan, 2008). BS conversion utilizes sodium BS to convert all unmodified cytosines to uracil by deamination but does not deaminate 5-mC or 5-hmC. The converted cytosines (C, 5-fC, or 5-caC) are read as thymines during sequencing, while the unconverted cytosines (5-mC or 5-hmC) are read as cytosines. From these data, the percent of methylation (beta value) at each cytosine can be calculated from the proportion of cytosines and thymines detected at each position (**Figure 1**).

Unfortunately, 5-mC and 5-hmC are both resistant to deamination during BS conversion, meaning BS-based methods are unable to differentiate between these two marks (Huang et al., 2010; Jin et al., 2010). As such, studies utilizing traditional BS treatment actually captured both 5-mC and 5-hmC, which may confound their identified associations between differential DNA methylation and transcriptional control. To address this issue, multiple technological advancements have allowed for specific profiling of 5-mC and 5-hmC at the base-pair level. Currently, there are two BS treatment-based methods used to measure 5-hmC levels—oxidative BS treatment (oxBS) and Tet-assisted BS treatment (TAB). More recently, additional novel techniques have been developed to estimate true 5-mC and true 5-hmC values, including APOBEC3A-mediated deamination sequencing, Tet-assisted pyridine borane sequencing, and AbaSI-sequencing (Sun et al., 2013; Li et al., 2018; Liu et al., 2019). These alternate methods hold promise but have not yet been widely adopted by the field. As such, this article focuses on a new statistical approach to deal with paired 5-mC and 5-hmC data from BS treatment-based methods.

### Bisulfite/Oxidative Bisulfite Treatment

Oxidative BS (oxBS) treatment involves chemically mediated selective oxidation of 5-hmC to 5-fC prior to BS conversion by potassium perruthenate (KuRO_4_). After this oxidation step, 5-hmC acts like 5-fC during BS conversion and is converted to uracil and read as thymine in subsequent sequencing reactions. 5mC remains unaffected by KuRO_4_, is not deaminated by BS, and is read as cytosine. Thus, oxBS provides a measure of 5-mC only (“true 5-mC”) (Booth et al., 2013) (**Table 1**). This method must be paired with traditional BS conversion, which provides a combined measure of 5-mC and 5-hmC, and an estimation step must be performed to generate an estimate of 5hmC. Currently, paired BS/oxBS is the most commonly used method to generate paired 5-mC and 5-hmC data; it is standard practice to use a maximum likelihood estimate (MLE) method to estimate 5hmC levels from paired BS/oxBS data (Xu et al., 2016).

**Table 1:** Beta value estimation for each described method for measuring base-pair resolution 5-hmC.

Beta value equation: β_mC_ + β_hmC_ + β_C_ = 1		
Method	Measured	Estimated by comparison to BS
BS	β_mC_ + β_hmC_	N/A
oxBS	β_mC_	β_hmC_
TAB	β_hmC_	β_mC_

The various DNA treatment methods described in the text—BS, oxBS, and TAB—allow for specific tagging and measurement of different DNA modifications. By comparing beta values generated from these methods with those from BS treatment data, β_hmC_ and β_mC_ can be estimated. As indicated by the equation at the top of the table, the sum of beta values for all DNA modifications is always equal to 1. This is because beta values represent the proportions of each modification, not measures of magnitude. As a result of being proportions, β_hmC_ and β_mC_ will always have values between 0 and 1.

### Tet-Assisted Bisulfite Treatment

TAB method is an enzyme-based method where 5-hmC is specifically protected from ten-eleven translocase (TET) enzyme-mediated oxidation. In this method, a β-glucosyltransferase enzyme is used to add a glucose moiety to 5-hmC prior to treatment with recombinant TET enzyme. The TET enzyme oxidizes 5-mC but not glucosylated 5-hmC, to 5-caC, a DNA modification that can be BS converted (Yu et al., 2012).

In essence, this method selectively protects 5-mC, leaving 5-hmC and the other modified cytosines available for BS conversion. As a result, TAB directly measures 5-hmC (“true 5-hmC”) at the base-pair level and can be performed without paired BS conversion (**Table 1**). However, the TAB method only measures 5-hmC, and does not provide any information on 5-mC. In addition, the enzymatic treatment required for TAB can be quite costly. Based on these considerations, use of the TAB method remains limited compared to BS/oxBS treatment.

### Generation of 5-Methylcytosine and 5-Hydroxymethylcytosine Beta Values

For genome-wide assessment of 5-mC and 5-hmC, each of the methods described previously can be paired with sequencing arrays (i.e., Illumina 450K/EPIC BeadChip), reduced representation sequencing, or whole-genome sequencing. Choosing between these available methods is not only a question of cost but also an experimental question, tissue type, and desired genomic coverage. Discussion of these specific issues is beyond the scope of this commentary; they are discussed in depth elsewhere (Sun et al., 2015; Kurdyukov and Bullock, 2016; Yong et al., 2016). The issues related to co-analysis of 5-mC and 5-hmC exist for all three types of data generation.

Following conversion of DNA by any of these three methods and subsequent analysis by sequencing arrays (i.e., Illumina 450K/EPIC BeadChip), reduced representation sequencing, or whole-genome sequencing, beta values for each modification can be calculated at each assayed cytosine. Beta values are ratios of modified (5-mC or 5-hmC) and unmodified (C) alleles, with values between 0 (unmodified) and 1 (fully modified); added together, the sum of these beta values at each cytosine equals 1 (**Table 1**).

## Problems in 5-Methylcytosine and 5-Hydroxymethylcytosine Data Analysis

Despite the significant technological advances in differentiating 5-mC and 5-hmC, standard statistical methods for co-analyzing 5mC and 5hmC do not yet exist. At an individual CpG site, both 5-mC and 5-hmC can contribute to gene regulation, but none of the available bioinformatics tools provide a function for co-analyzing 5-mC and 5-hmC β values. As a result, existing studies have focused on either examining the distribution of 5-hmC across the genome in isolation (Green et al., 2016; Johnson et al., 2016; Hernandez Mora et al., 2018) or treating 5-mC and 5-hmC β values as independent variables, analyzing each epigenetic mark as a separate dataset to identify differentially methylated and hydroxymethylated regions (Glowacka et al., 2018; Zhang et al., 2018). While there is utility to both of these approaches, the results are difficult to reconcile into a clear picture of the underlying biology for two main reasons: 1) the methodological and biological interdependence of 5-mC and 5-hmC and 2) the different distributions of β_mC_ and β_hmC_. This uncertainty complicates functional interpretation of BS-based DNA modification data, since 5-mC and 5-hmC have distinct genomic distributions and regulatory functions (Shen and Zhang, 2013; Skvortsova et al., 2017). Furthermore, this type of differential DNA modification misclassification is particularly relevant in nervous system tissue, where 5-hmC is present at high levels (Globisch et al., 2010; Szwagierczak et al., 2010; Nestor et al., 2012; Cheng et al., 2015). Later, we run through these concerns in greater detail and, then, propose a statistical method for co-analyzing paired 5-mC and 5-hmC levels.

### Interdependence of 5-Methylcytosine and 5-Hydroxymethylcytosine

After measuring genome-wide 5-mC and 5-hmC at the base-pair level, a simple approach would be to split these two epigenetic marks into separate datasets for analysis. While this method is attractive, it fails to account for the interdependence of 5-mC and 5-hmC data. These two epigenetic marks are often related to each other biologically and methodologically. Biologically, 5-hmC is produced through direct oxidation of 5-mC (Shen et al., 2014), meaning 5-hmC β values are directly dependent on 5-mC β values. In addition to their biological relationship, 5-mC and 5-hmC β values generated from BS/oxBS experiments are also methodologically related, since calculation of 5-hmC is dependent upon either subtraction or a maximum likelihood estimation step (Booth et al., 2013; Houseman et al., 2016; Xu et al., 2016). Unless one was to measure 5-mC and 5-hmC directly through an alternative combination of the presented techniques, this methodological interdependence is unavoidable. Modeling approaches that treat 5-mC and 5-hmC β values as independent variables do not account for this inherent interdependence and limit one’s ability to comprehensively identify regions where 5-mC and 5-hmC have differential responses to an experimental condition.

### Differential Distributions of **β**_mC_ and **β**_hmC_

**Figure 2:**
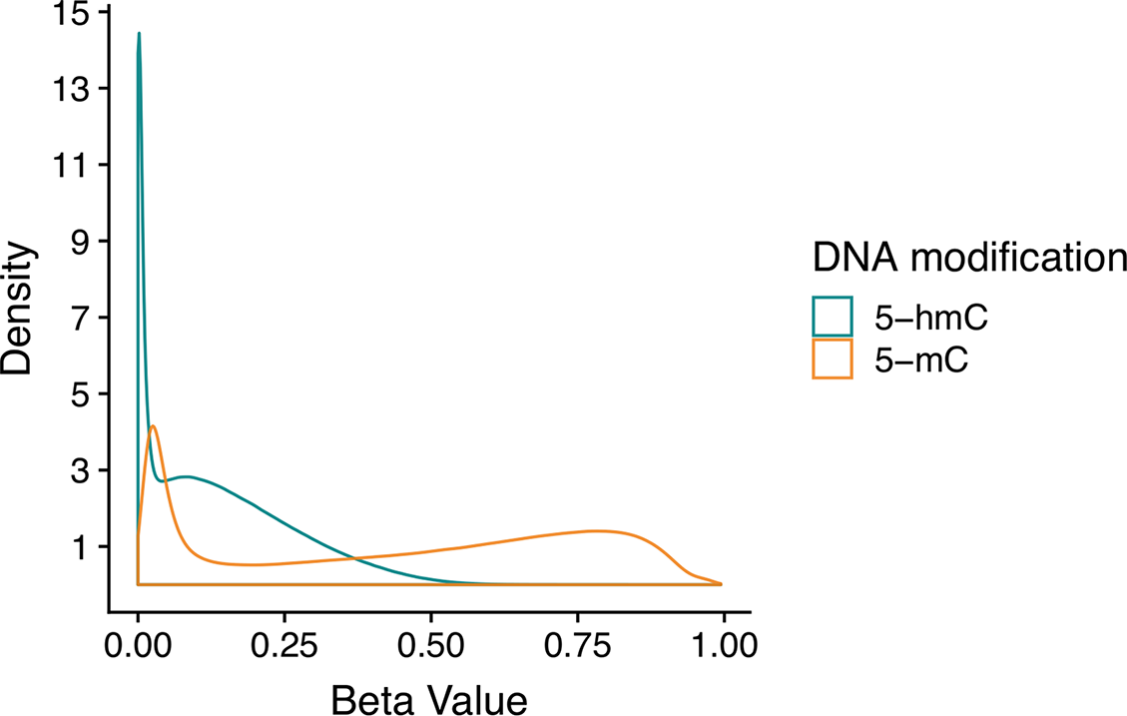
Beta value distributions for 5-mC and 5-hmC from example BS-EPIC/oxBS-EPIC array data. Beta values for 5-mC and 5-hmC were estimated from in-house example BS/oxBS-EPIC data using the oxBS.MLE function with default parameters in the *ENmix* R package. Since many CpG sites have appreciable 5-mC, but no 5-hmC, estimated 5-hmC beta values are zero-enriched after maximum likelihood estimation from BS/oxBS data.

Even in the brain, where 5-hmC is present at comparatively high levels, it is still a rare event. Thus, many CpG sites have appreciable 5-mC but no 5-hmC, which means that estimated 5-hmC β values are zero-enriched (**Figure 2**). On a genome-wide scale, 5-mC has a beta distribution, and 5-hmC has a zero-inflated beta distribution. Given these divergent distributions, independent tests for differential 5-mC and 5-hmC need to utilize specific statistical approaches that include appropriate assumptions for their distributions. When different statistical tests are used for 5-mC and 5-hmC, the results from differential testing are difficult to reconcile. Furthermore, zero values for 5-hmC are typically estimated from paired BS/oxBS data, so it can be difficult to determine whether 5-hmC β values are true biological zeroes or technical artifacts of the data generation method. This complicates the downstream identification of treatment-induced active demethylation at specific genomic regions.

### Scenarios Where Independent Analysis Breaks Down

Modeling 5-mC and 5-hmC data separately requires a larger number of statistical tests than analyzing a single dataset. This increases the risk for false positives and may impede accurate interpretation of the data. While multiple testing correction methods can be used to address this concern, these statistical techniques can drastically limit one’s ability to detect true positives, especially in studies with a small sample size. As a result, analyzing 5-mC and 5-hmC data using separate models could negatively impact the ability of a project to identify regions of differential methylation and hydroxymethylation.

**Figure 3:**
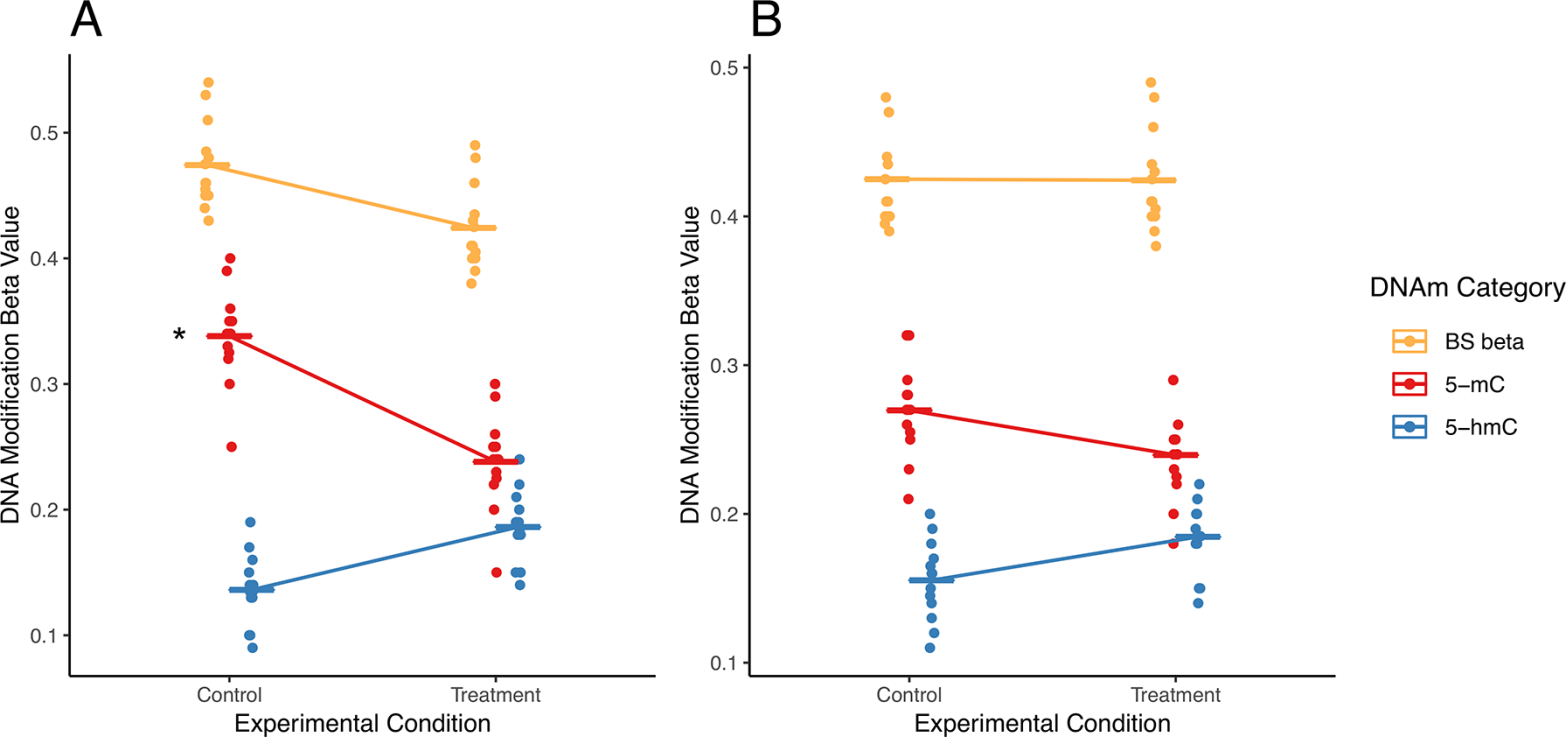
Examples of simultaneous, treatment-related changes in 5-mC and 5-hmC. Here, we use mock data to present two hypothetical scenarios in which experimental condition alters levels of 5-mC and/or 5-hmC in brain tissue. **(A)** In the first example, 5-mC significantly decreases, and 5-hmC shows a nonsignificant increase at a CpG site, while combined levels of DNA modifications decrease in exposed compared with those in control. Using a “traditional” modeling approach with separate models for each DNA modification, only the significant change in 5-mC would be identified (as indicated by asterisk), whereas the corresponding shift in 5-hmC at this CpG would not be identified. Our proposed interaction term model would identify this CpG as a site where there is a shift in the balance between 5-mC and 5-hmC. As such, our proposed analysis would supplement the information produced by the traditional model. **(B)** In the second example, 5-mC shows a nonsignificant decrease, and 5-hmC shows a nonsignificant increase at a CpG site; meanwhile, combined DNA modifications remain the same by experimental group. Using a “traditional” modeling approach with separate models for each DNA modification, this CpG would not be identified as significant for either epigenetic mark. However, depending on the statistical power of our test, our proposed interaction term model could identify this CpG as a site where there is a shift in the balance between 5-mC and 5-hmC. As a result, our proposed analysis would provide additional information about the subtle shifts in these epigenetic marks at this CpG site. The asterisk indicates a significant change by experimental condition that would be identified using separate models for each DNA modification.

In addition to potential statistical errors, there are multiple scenarios in which independent analysis of 5-mC and/or 5-hmC could fail to capture a complete picture of differential DNA modifications (**Figure 3**). Here, we present two potential scenarios in which independent analysis of 5-mC and 5-hmC presents limitations to the biological interpretation of the results.

In a first hypothetical scenario, the total proportion of modified cytosines decreases at a given CpG site, but only one modification is identified as statistically significant, leading to an incomplete view of the underlying biology (**Figure 3A**). In the specific example provided, the proportion of 5-mC significant decreases, and the proportion of 5-hmC shows a nonsignificant increase. These example data suggest oxidation of 5-mC to 5-hmC at the measured CpG site. This oxidative processing may be part of active demethylation, which would lead to the observed decrease in total DNA modifications. However, downstream statistical analysis that treats 5-mC and 5-hmC as independent measures would only pick up the significant changes in 5-mC and would likely not identify the corresponding directional shift in 5-hmC. As a result, the selected analysis approach could lead to improper biological interpretation of the results.

In a second scenario, 5-mC shows a nonsignificant decrease, and 5-hmC shows a nonsignificant increase; meanwhile, combined DNA modifications remain the same by experimental group (**Figure 3B**). These data suggest a region with subtle oxidative processing of 5-mC to 5-hmC, but this shift in DNA modifications would not be detected in downstream statistical analysis that treats 5-mC and 5-hmC as independent measures.

For the described hypothetical scenarios, changes in the balance between 5-mC and 5-hmC at a measured CpG site may not be detected if the individual DNA modifications were analyzed as independent datasets. These dynamic regions of active DNA modification cycling may play an important biological role and should not be ignored. To address these concerns, researchers need a method to simultaneously analyze 5-mC and 5-hmC levels; unfortunately, no such statistical method currently exists in the literature.

## Potential Solutions

### Measuring True 5-Methylcytosine and 5-Hydroxymethylcytosine

One way to address some of the statistical concerns brought up in the previous section would be to measure true levels of 5-mC and 5-hmC. For example, given that TAB and oxBS treatment selectively measure 5-hmC and 5-mC, these methods could be combined to measure true values for 5-hmC and 5-mC. This approach would bypass the required estimation step used to calculate 5-hmC levels in BS/oxBS experiments, thereby reducing the methodological interdependence of 5-mC and 5-hmC. However, this type of combined approach does not address the statistical concerns laid out previously. Furthermore, the TAB and oxBS method are reliant upon BS conversion, which negatively impacts DNA quality. This loss of sample integrity could complicate integration of data generated from TAB and oxBS experiments. Ideally, further work in the field will lead to development of reliable methods to measure 5-mC and 5-hmC directly and independently without a harsh BS conversion step to allow for consistent genomic coverage.

### Statistical Methods to Analyze 5-Methylcytosine and 5-Hydroxymethylcytosine as Related Measures

**Figure 4:**
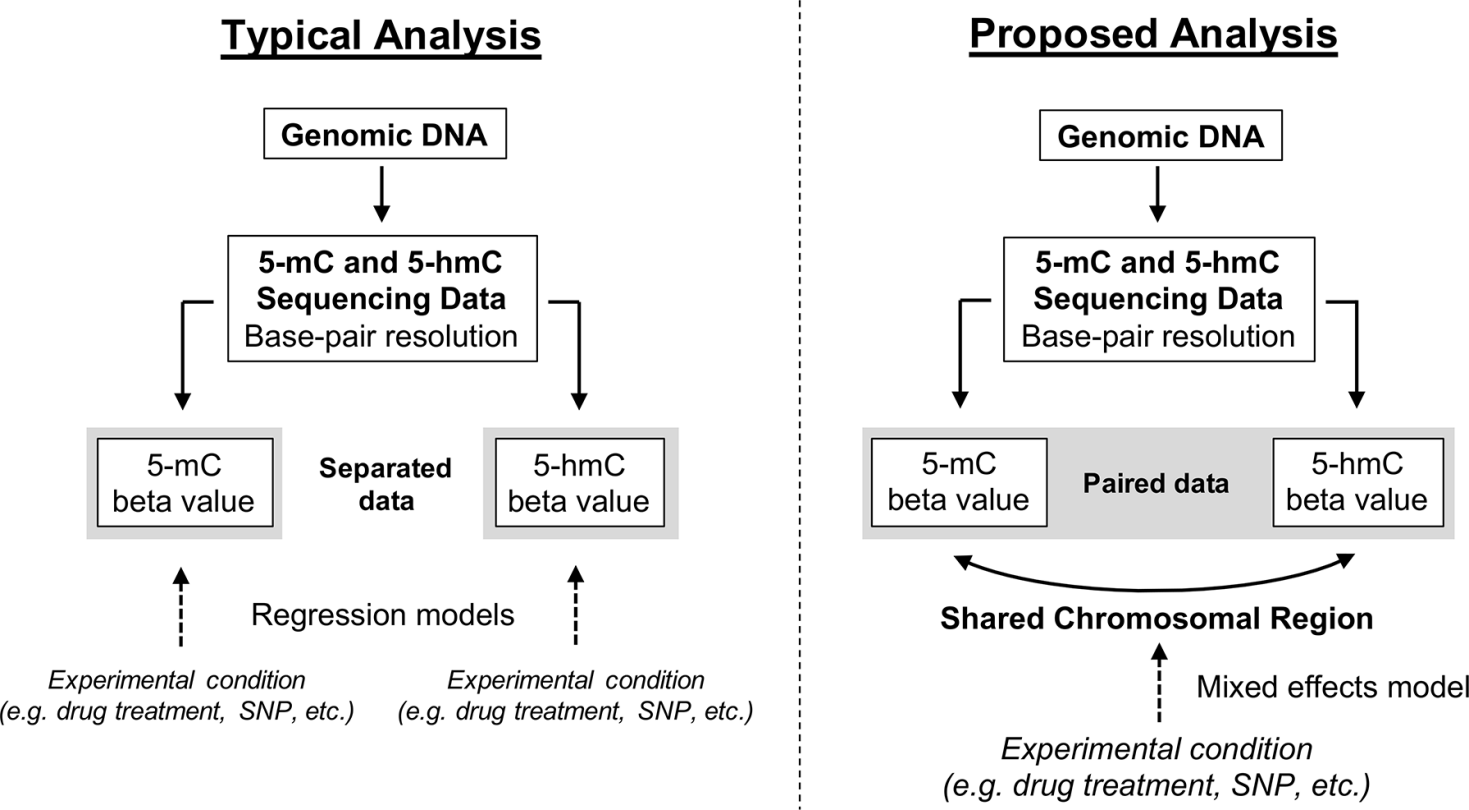
Conceptual framework for reconciling 5-mC and 5-hmC data using a mixed effects modeling approach. Upon generating base-pair resolution 5-mC and 5-hmC data, the current analysis approach is to run separate regression models on the beta values for 5-mC and 5-hmC. However, this approach fails to account for the inherent dependence of 5-hmC on 5-mC and limits a researcher’s ability to quickly and comprehensively identify regions where 5-mC and 5-hmC have differential responses to an experimental condition. Here, we propose an alternative ME modeling approach in which the 5-mC and 5-hmC beta values are treated as repeated measures. Using an interaction term in our proposed model, it will be possible to identify regions where the directionality of response to experimental condition changes by DNA modification (5-mC vs. 5-hmC). This approach is not necessarily a replacement for separate regression models but rather a supplement.

Here, we propose a new approach for modeling paired 5-mC and 5-hmC data (**Figure 4**). Rather than treating β_mC_ and β_hmC_ as independent variables, we propose treating these two data points as “repeated” measures of a single outcome variable—”DNA modification.” It is important to note that 5-mC and 5-hmC levels are separate epigenetic marks and do not represent true biological repeats. However, as outlined previously, 5-mC and 5-hmC are both measured at each CpG site, and the beta values for these two marks are dependent upon each other both biologically and statistically. To account for this relationship in statistical terms, we propose a mixed effects (ME) modeling approach (Laird and Ware, 1982). Under this approach, each model would include a fixed effect for experimental condition/group and random effects for CpG probe ID and batch to account for within-site and within-batch variability. Given that only two data points (5-mC and 5-hmC beta values) are included in each model *per* sample, inclusion of a random effect for CpG probe ID also accounts for within-sample variability. To determine the differential effects of an experimental condition on 5-mC and 5-hmC, an interaction term between experimental condition and a categorical DNA modification variable (DNA_mod_cat: “5-mC” or “5-hmC”) would also be included in the ME model. This interaction term determines whether the direction of the relationship between β values and experimental condition varies by DNA modification category (5-mC/5-hmC).

Mixed models could be fit using a model in the following form:

*y = β* *_0_* *+ β* *_1_* *x* *_1_* *x* *_2_* *+* *β* *_2_* *x* *_3_* *+…+ b* *_1_* *x* *_i_* *+ b* *_2_* *x* *_j_* *+ ε*
*y* = 5-mC or 5-hmC beta value
*β_0_* = Intercept
*x_1_* = Experimental condition (e.g., disease status, exposure)
*x_2_* = DNA modification (5-mC or 5-hmC) categorical variable
*x_3_* = Sex
*x_i_* = ID
*x_j_* = Batch
ε = error term

In this model, β is used for fixed effect term coefficients, whereas *b* is used for random effect coefficients. The ellipses refer to the fact that additional covariates could be added to the model.

Alternatively, the main effect of experimental condition on DNA modifications could be tested using a model in the following form:

*y = β* *_0_* + *β* *_1_* *x* *_1_* *+* *β* *_2_* *x* *_2_* *+…+ b* *_1_* *x* *_i_* *+ b* *_2_* *x* *_j_* *+ ε*
*y* = 5-mC or 5-hmC beta value
*β* *_0_* = Intercept
*x* *_1_* = Experimental condition (e.g., disease status, exposure)
*x* *_2_* = Sex
*x* *_i_* = ID
*x* *_j_* = Batch
ε = error term

Once again, β is used for fixed effect term coefficients, whereas *b* is used for random effect coefficients. The ellipses refer to the fact that additional covariates could be added to the model.

**Figure 5:**
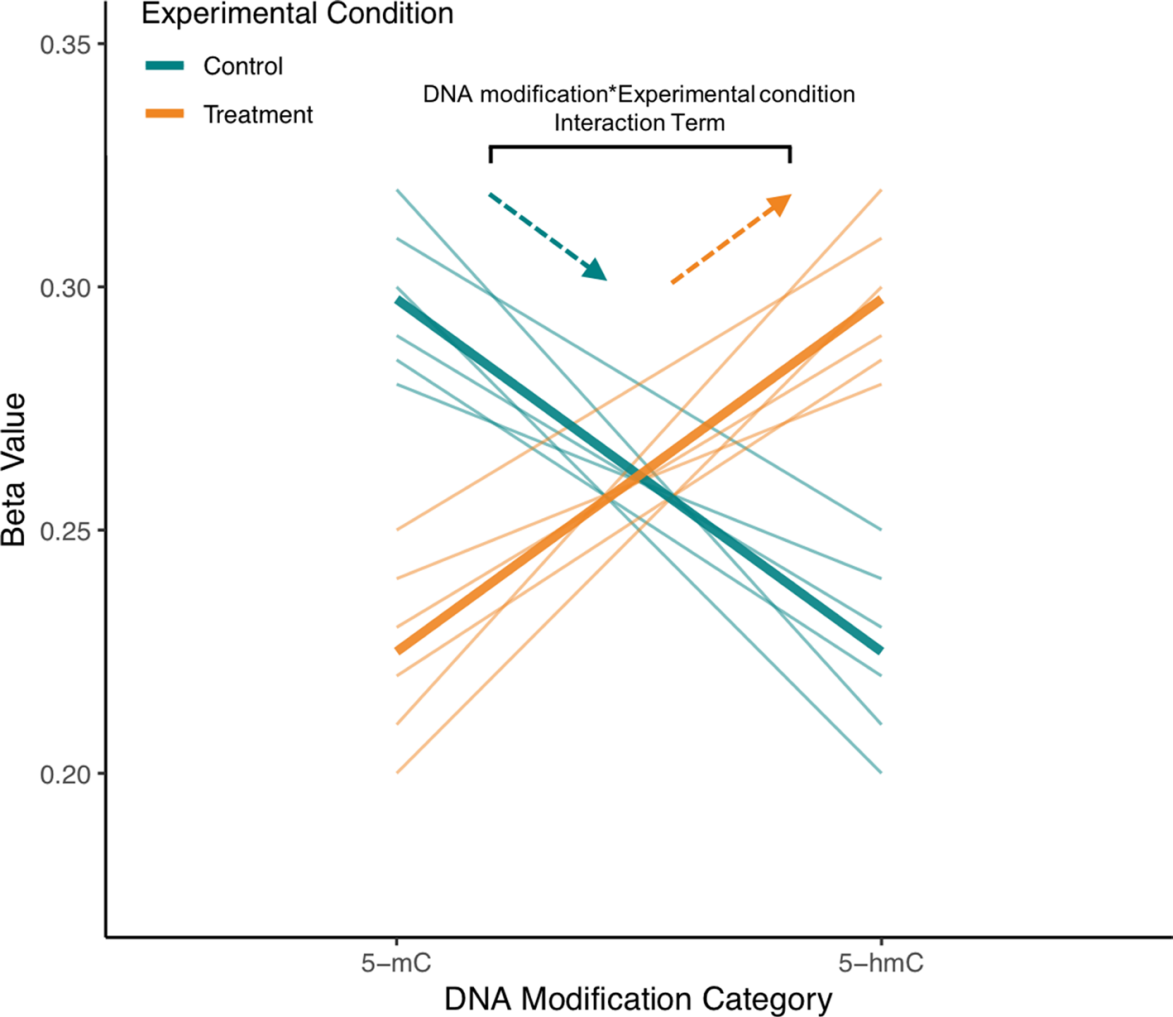
Visualization of interaction term from example repeated measures model for single CpG site from mock BS/oxBS data. In the proposed ME model treating 5-mC and 5-hmC as repeated measures, a random effect for ID will account for the correlation between 5-mC and 5-hmC at a CpG site. Meanwhile, a “DNA modification*Experimental Condition” interaction term will be used to determine whether 5-mC and 5-hmC differ in their response to experimental condition. In the visualized mock data, brain samples from exposed animals have an increased slope compared with those from control animals, indicating that experimental treatment is shifting the CpG site toward 5-hmC in the brain. As indicated in the figure, this difference in slope is modeled by the “DNA modification*Experimental Condition” (뭐β*_1_* *x* *_1_* *x* *_2_*) interaction term. The proposed statistical approach can pick up regions where trajectories of 5-mC/5-hmC change while also accounting for the fact that 5-mC and 5-hmC are dependent measures. Furthermore, fixed effect terms for 5-hmC and 5-mC could also be included to model the response of either 5-mC or 5-hmC to experimental treatment.

The modeling approach outlined earlier should be applicable to all types of paired 5-hmC and 5-mC data, provided the data structure is quantitative and at base-pair resolution. In addition, this approach should be appropriate for various analysis methods, provided they allow for a ME design. Previous work has shown that beta regression (BR) and ratio of correlated gammas (RCG) modeling approaches are appropriate for detecting methylation differences on a genome-wide scale and have greater specificity than linear models fitted to raw or normalized beta values, especially for group sizes less than 500 (Triche et al., 2016; Weinhold et al., 2016; Mansell et al., 2019). As such, fitting these types of models according to the repeated measures design outlined earlier should allow for simultaneous analysis of paired 5-mC and 5-hmC data, despite their potential differences in beta value distributions. Inclusion of an interaction term in the proposed model captures the potential transition from 5-mC to 5-hmC, allowing researchers to investigate whether experimental variables have distinct effects on 5-mC and 5-hmC dynamics at specific CpG sites/regions in neuronal tissue (see example in **Figure 5**). However, inclusion of an interaction term complicates interpretation of the main effect of experimental condition on the outcome of interest (i.e., DNA modification beta value). As a result, ME models with an individual term for experimental condition, but no interaction term, can be used to model the response of either 5-mC or 5-hmC to experimental treatment. As the number of individual CpGs being tested increases, researchers must also consider instituting corrections for multiple testing—e.g., Benjamini–Hochberg false discovery rate (Benjamini and Hochberg, 1995). Future bioinformatics tools that aim to co-analyze paired 5-mC and 5-hmC data should implement this type of statistical approach on a genome-wide scale. This is particularly critical for epigenetics studies in brain tissue, where 5-hmC is both abundant and functionally relevant.

## Implementation of Proposed Modeling Approach

To test our proposed approach, we sourced recently published data from an Alzheimer’s disease (AD) study to test our proposed statistical analysis method on real data (Smith et al., 2019). At the same time, we also analyzed the data using the “traditional” approach—modeling the effect of disease status on 5-mC and 5-hmC using separate BR models and, then, checking for overlap between the lists of differentially methylated and differentially hydroxymethylated probes. By comparing the outputs from our new approach and the traditional approach, we aimed to provide a proof of concept that our new approach is able to detect additional regions of both differential DNA methylation and hydroxymethylation.

### Methods

In the selected study, Illumina 450K DNA methylation array data were generated from human brain tissue (Smith et al., 2019) (Gene Expression Omnibus accession: GSE105109). Given that this was a proof of concept, we limited our analysis to only one tissue (entorhinal cortex), one sex (male), and only control or Braak stage VI brains to limit covariates in this preliminary test of our modeling approach. In total, we analyzed BS/oxBS-450K data from 14 control and 22 AD entorhinal cortex samples.

**Figure 6:**
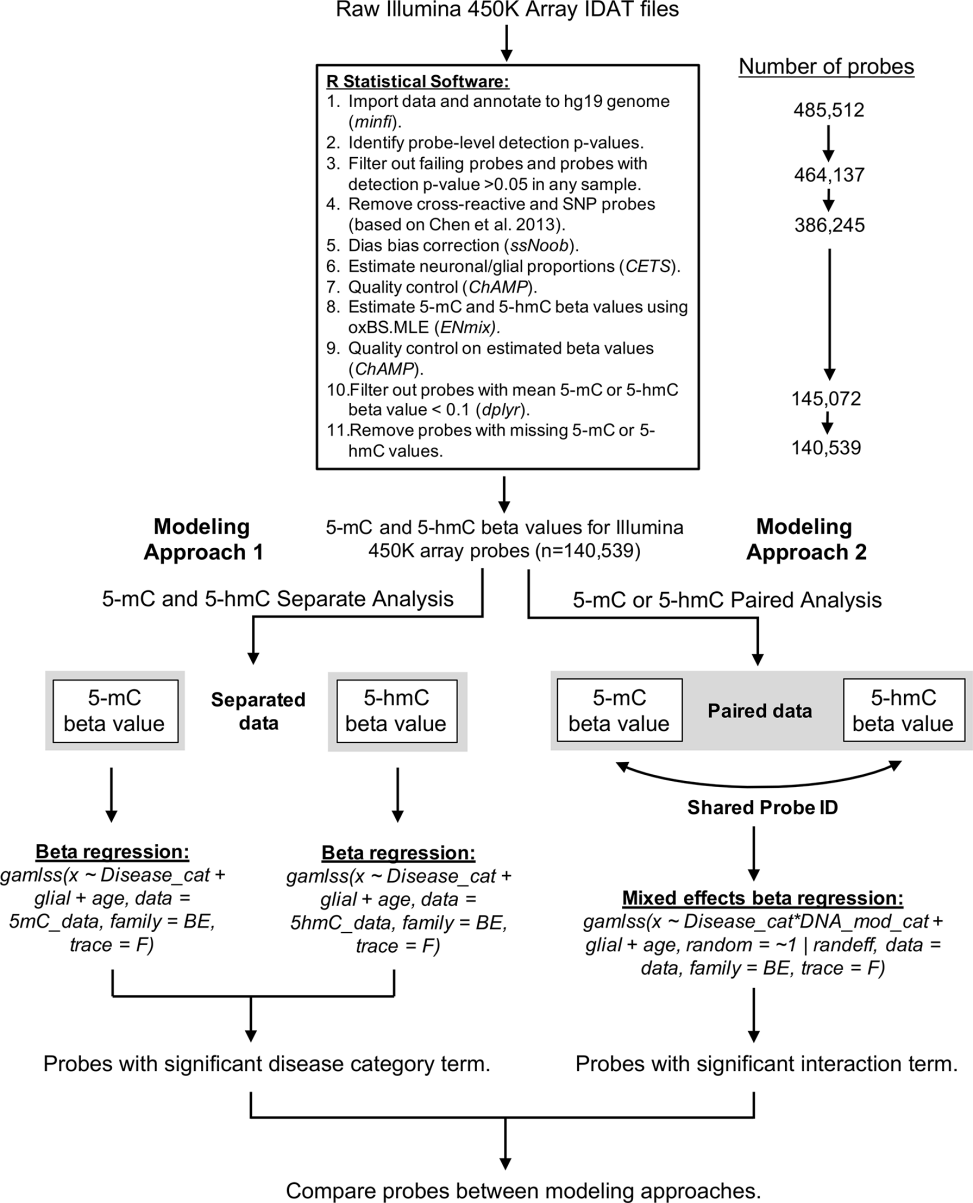
Bioinformatics pipeline for oxBS-450k analyses. The described bioinformatics pipeline was used to perform both paired and parallel modeling of 5-mC/5hmC by AD status. Publicly available Illumina 450K array IDAT files were sourced from Gene Expression Omnibus accession GSE105109 (Smith et al., 2019). All data processing and analysis were performed using R statistical software (version 3.5.3).

A custom bioinformatics pipeline was developed in R to estimate proportions of 5-mC and 5-hmC in each sample (**Supplementary File 1**). This pipeline combined the *minfi* (version 1.22.1), *ChAMP* (version 2.14.0), and *ENmix* (version 1.12.4) packages in R (**Figure 6**). Quality control was assessed for internal control probes using the *ENmix* plotCtrl function. Probes were first filtered based on a detection p-value > 0.05 in any sample. Out of 485,512 probes included on the Illumina 450k array, the detection p-value cutoff filtered out 21,375 probes. In addition, one control sample was excluded due to a high percentage (>10%) of failed probes, leaving the control group with a sample size of 13. Cross-reactive probes and probes containing single-nucleotide polymorphisms were removed based upon previous identification (Chen et al., 2013). This process removed an additional 77,892 probes from the samples. A comparison of technical replicates revealed consistent results across arrays. After removal of technical artifacts, dye-bias correction was performed with *ssNoob* within *minfi* (Fortin et al., 2017). The proportion of neuronal vs. glial cells in each sample was estimated with *CETS* (Guintivano et al., 2013). The oxBS.MLE function in the *ENmix* package was used to calculate MLEs of 5-mC and 5-hmC beta values for each probe (Xu et al., 2016), and batch effects were assessed using the *ChAMP* package (Tian et al., 2017). After beta value estimation, we filtered out samples where mean 5-mC or 5-hmC beta value < 0.1; this step removed an additional 241,173 probes. The beta value > 0.1 cutoff was selected based on its use in the data’s original study (Smith et al., 2019) and removes the issue of zero-inflation for 5-hmC beta values in the dataset. As a final step, we removed probes that had any missing 5-mC or 5-hmC values to ensure appropriate modeling; this removed an additional 4,533 probes, leaving us with 140,539 probes in the final analysis.

For differential DNA modification analyses, we utilized two approaches to model the effect of AD on 5-mC and 5-hmC data (**Figure 6**). In the first, “traditional” modeling approach, 5-mC and 5-hmC beta values were treated as independent variables and analyzed separately using BR in the *gamlss* R package (Rigby and Stasinopoulos, 2005). This is similar to the method employed by Smith et al. (2019) in their original publication, except they used linear regression models that corrected for age, sex, and neuron/glia proportion. We elected to run our own separate analyses here, rather than use the published analysis performed by Smith et al., because our paired analysis uses a specific R package (*gamlss*) that allows for the use of BR. Furthermore, we selected only a subset of the publicly available data to analyze, which meant that our models had fewer covariates than the original publication. Separate models were fit for each processed probe according to the following formulas in R:

$$\begin{array}{l} \mathrm{gamlss}\;(5-\mathrm{mC}\;\mathrm{beta}\;\mathrm{value}\sim \mathrm{Disease}\;\mathrm{status}+\mathrm{Glial}\;\mathrm{proportion}+\\ \;\mathrm{Age},\mathrm{data}=5\mathrm{mC}.\mathrm{data},\;\mathrm{family}=\mathrm{BE},\;\mathrm{trace}=F)\\ \mathrm{gamlss}\;(5-\mathrm{hmC}\;\mathrm{beta}\;\mathrm{value}\sim \mathrm{Disease}\;\mathrm{status}+\mathrm{Glial}\;\mathrm{proportion}+\\ \;\mathrm{Age},\mathrm{data}=5\mathrm{mC}.\mathrm{data},\;\mathrm{family}=\mathrm{BE},\;\mathrm{trace}\;=\;F) \end{array}$$

In the second, novel modeling approach, we treated the 5-mC and 5hmC beta values as “repeated” measures of a single outcome variable—“DNA modification.” To achieve this in statistical terms, we used BR combined with an ME modeling approach (Laird and Ware, 1982). The ME models included a fixed effect for AD status and a random effect for probe ID to account for within-probe variability. Based on a lack of batch effects in CHAMP R package QC, batch was not included as a random effect in the mixed models. To determine the differential effects of AD status on paired 5-mC and 5-hmC, we included an interaction term between disease status and categorical DNA modification variable (two values: “5-mC” or “5-hmC”). Mixed models were fit according to the following formula:

$$\begin{array}{l} \mathrm{gamlss}(5\text{-}\mathrm{mC}\;\mathrm{and}\;5\text{-}\mathrm{hMC}\;\mathrm{beta}\;\mathrm{value}\sim \mathrm{Disease}\;\mathrm{status}*\\ \mathrm{DNA}\;\mathrm{modification}\;\mathrm{category}+\mathrm{Glial}\;\mathrm{proportion}+\mathrm{Age}+\text{[}1|\mathrm{ID}\text{]},\;\\ \mathrm{data}=5\mathrm{mC}.5\mathrm{hmC}.\mathrm{data},\;\mathrm{family}=\mathrm{BE},\;\mathrm{trace}=F) \end{array}$$

Correction for multiple testing was performed on p-values from all BR modeling using the p.adjust function in the R *stats* package. Within the p.adjust function, we selected “fdr,” which utilizes the Benjamini–Hochberg false discovery rate (FDR) method for multiple testing correction (Benjamini and Hochberg, 1995). All statistical models were run using R statistical software (version 3.5.3). Annotation of detected differential probes was performed using the Illumina 450K array manifest. QC plots, lists of differential probes, and code for raw data processing, filtering, and modeling are available as **Supplementary Material**.

### Results

In the first, “traditional” modeling approach, genome-wide differential methylation and hydroxymethylation by AD were assessed using separate BR models for 5-mC and 5-hmC data. In the separate models, we identified only two CpG probes—cg24998879 and cg05272827—that showed a significant increase in 5-mC in Alzheimer’s cortex compared to control (FDR < 0.10). We also identified a single CpG probe—cg02253760—that showed a significant increase in 5-hmC in Alzheimer’s cortex compared with that in control (FDR < 0.10). These data were similar to the results reported in the dataset’s original publication, where the authors identified only one differentially methylated probe (DMP) 2 differentially methylated regions, and one differentially hydroxymethylated region (Smith et al., 2019). Since there was so little significance in the separate models, it was difficult to compare the traditional method with our novel approach, so we repeated the separate modeling approach with a more lenient p-value < 0.001 cutoff. Using this new cutoff, we identified 232 DMPs and 568 differentially hydroxymethylated probes (DHMPs) (**Table 2**). In the DMPs, 214 were hypermethylated (91.8%), and 19 were hypomethylated (8.2%). In the DHMPs, 461 probes were hypo-hydroxymethylated (81.2%), and 107 were hyper-hydroxymethylated (18.8%). The DMPs annotated to 183 genes, and the DHMPs annotated to 373 genes (**Table 2**). Comparing the significant DMPs and DHMPs by chromosomal position, 68 probes showed both significant differential DNA methylation and hydroxymethylation with AD.

**Table 2:** Significant probes in parallel and paired modeling approaches. In the paired modeling approach, genome-wide differential methylation and hydroxymethylation by Alzheimer’s disease were simultaneously assessed using a mixed effects beta regression model for paired 5-mC and 5-hmC data. An interaction term between disease status and DNA modification category was used to co-analyze changes in 5-mC and 5-hmC by disease status. In this paired model, we identified 14,183 probes showed a significant interaction between Alzheimer’s disease status and DNA modification category (FDR < 0.05). In the second, parallel modeling approach, we assessed differential 5-mC and 5-hmC by Alzheimer’s disease using separate beta regression models. In this second approach, we instituted a more lenient p-value cutoff < 0.001 for significance calling. Using this cutoff, we identified 232 probes that showed differential methylation and 568 probes that showed differential hydroxymethylation by Alzheimer’s disease status. “Negative” and “positive” beta coefficients refer to the directionality of the effect estimate for beta regression modeling terms. Genes were annotated using the Illumina 450k DNA methylation array manifest; total annotations for significant probes are shown in the tables on the right.

AD-related 450K probes				Annotated Gene IDs		
	Separate Modeling				Separate Modeling	
Disease Status Beta Coefficient	5-mC	5-hmC		Disease Status Beta Coefficient	5-mC	5-hmC
Negative	19	461		Negative	14	305
Positive	213	107		Positive	169	75
Total	232	568		Both	0	7
*Beta coefficients for AD vs. control samples; adjusted for neuron/glial proportion and age; p-value < 0.001				Total unique IDs	183	373
				*Beta coefficients for AD vs. control samples; adjusted for neuron/glial proportion and age; p-value < 0.001		
AD-related 450K probes				Annotated Gene IDs		
	Paired Modeling				Paired Modeling	
Interaction Term Beta Coefficient	5-mC + 5-hmC			Interaction Term Beta Coefficient	5-mC + 5-hmC	
Negative	13,270			Negative	6,009	
Positive	913			Positive	607	
Total	14,183			Both	315	
*Beta coefficients for Disease*DNA modification category term; adjusted for neuron/glial proportion and age; FDR< 0.05				Total unique IDs	6,301	
				*Beta coefficients for Disease*DNA modification category term; adjusted for neuron/glial proportion and age; FDR < 0.05		

In the second, novel modeling approach, genome-wide differential methylation, and hydroxymethylation by AD were simultaneously assessed using an ME BR model for paired 5-mC and 5-hmC data. An interaction term between disease status and DNA modification category was used to co-analyze changes in 5-mC and 5-hmC by disease status. In this paired model, we identified 14,183 probes that showed a significant interaction between AD status and DNA modification category (FDR < 0.05), suggesting widespread shifts in the balance between 5-mC and 5-hmC by AD (**Table 2**). For the remainder of this paper, we will refer to these probes as differential interaction probes (DIPs). Within the DIPs, 13,270 had a negative interaction term beta coefficient (93.6%), and 913 had a positive interaction term beta coefficient (6.4%). A positive interaction term indicates an increase in the slope between 5-mC and 5-hmC in AD brains compared with that in control, which represents a shift toward a greater proportion of 5-hmC at a given CpG (see **Figure 5** for example). A negative interaction term indicates a decrease in the slope between 5-mC and 5-hmC in AD brains compared with that in control, which represents a shift toward a greater proportion of 5-mC at a given CpG. The DIPs annotated to 6,301 genes (**Table 2**). This large number of annotated genes suggests that an even more stringent FDR cutoff may be appropriate for this novel modeling approach. Furthermore, additional verification and biological confirmation of the proposed method is warranted.

### Comparison of Proposed Method With “Traditional” Analysis

Of note, all 68 of the overlapping probe IDs from the separate models were also significant in our interaction term modeling, indicating that the interaction term modeling was able to identify all regions of overlap from the separate modeling approach. In addition, of the 664 probes that were identified as significant in only one DNA modification (5-mC: n = 164; 5-hmC: n = 500), 610 (91.9%) were also identified in our interaction term modeling (5-mC: n = 127; 5-hmC: n = 483). After taking this overlap between statistical methods into account, 13,505 probes were only identified in the interaction term modeling, and 54 probes were only identified in the traditional analysis. These results indicate that our proposed model supplements the traditional approach. Using only separate models, we would have missed 13,505 probes that showed a significant shift in the balance between 5-mC and 5-hmC with AD. Furthermore, 610 probes that were only significant at a single DNA modification in the traditional analysis were also detected by our proposed analysis approach. As such, using only traditional modeling, we would have had an incomplete understanding of the changes occurring at these probes. These types of gaps in data could have profound effects on the conclusions drawn about the specific epigenetic changes occurring at each cytosine. In particular, the separate analysis seems to underestimate shifts between 5-mC and 5-hmC, instead focusing on differences between each of these marks and unmodified cytosines. In contrast, the paired analysis suggests that there are widespread, subtle shifts in the balance between 5-mC and 5-hmC in AD brain. By expanding on the traditional statistics used in neuroepigenetics, our novel modeling approach may improve the field’s understanding of how 5-mC and 5-hmC are altered in disease states.

**Figure 7:**
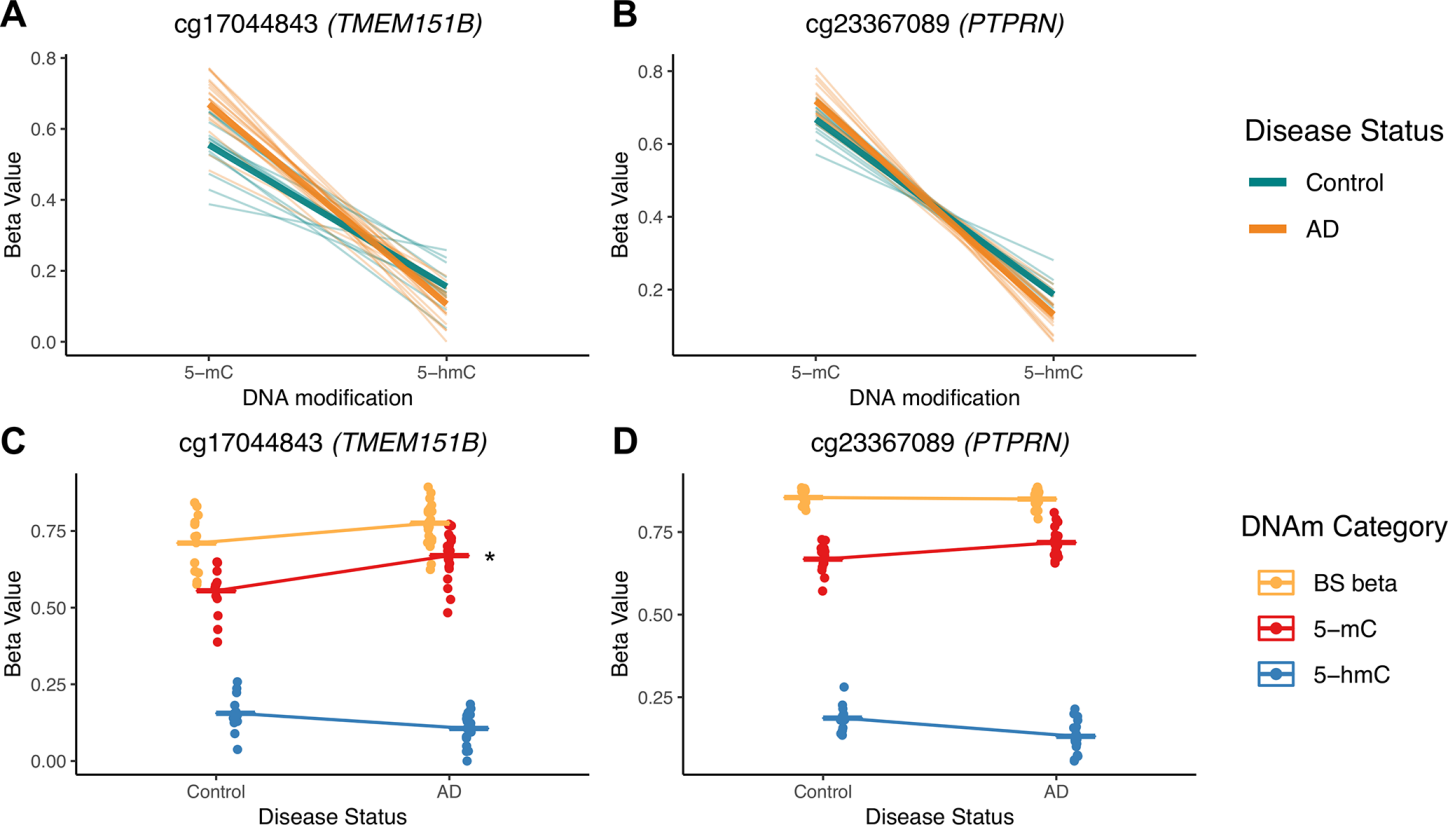
5-mC and 5-hmC levels at two example DIPs. To illustrate the value of our statistical approach, we visualized two DIPs at probes in the *TMEM151B* (cg17044843) and *PTPRN* (cg23367089) genes. Beta values for these two probes are represented in two ways—with either DNA modification category **(A**, **B)** or disease status **(C**, **D)** on the x-axis. Beta values are presented for raw BS converted DNA (BS beta), as well as 5-mC and 5-hmC, as calculated using maximum likelihood estimation. These two probes were selected to represent two scenarios in which traditional modeling misses information about 5-mC and/or 5-hmC, as previously outlined in **Figure 3**. At the cg17044843 probe, we found a significant negative interaction between DNA modification category and disease status **(A)**, but only 5-mC levels were identified as significant in traditional modeling **(C)**. Meanwhile, at the cg23367089 probe, we found a significant negative interaction between DNA modification category and disease status **(B)**, but neither 5-mC nor 5-hmC were significantly changed with AD in the traditional modeling approach **(D)**. At both of these sites, traditional modeling failed to capture subtle changes in 5-mC and/or 5-hmC levels with AD. In A and B, thin lines represent a slope between 5-mC and 5-hmC within individuals, and thicker lines represent smoothed means for the two disease status groups. In B and C, dots represent individuals, and lines represent magnitude and direction of changes between disease groups, not slope within individuals. The asterisk indicates a significant effect of AD on mean DNA modification beta value in traditional, separate modeling.

To illustrate potential advantages of this approach, we visualized two DIPs that illustrate the scenarios outlined in **Figure 3**; these probes are in the *TMEM151B* (cg17044843) and *PTPRN* (cg23367089) genes (**Figure 7**). For these two probes, beta values from raw BS converted DNA (BS beta), 5-mC, and 5-hmC, as calculated using maximum likelihood estimation are represented in two ways—with either DNA modification category (**Figures 7A, B**) or disease status (**Figures 7C, D**) on the x-axis. We visualized beta values for raw BS converted DNA (BS beta), 5-mC, and 5-hmC, as calculated using maximum likelihood estimation. At the cg17044843 probe, we found a significant negative interaction between DNA modification category and disease status, but only 5-mC levels showed a significant effect of AD in traditional modeling. Meanwhile, at the cg23367089 probe, we again found a significant negative interaction between DNA modification category and disease status, but neither 5-mC nor 5-hmC was significantly changed with AD in the traditional modeling approach. At both of these DIPs, traditional modeling failed to capture subtle changes in the balance between 5-mC and 5-hmC that occur with AD. While it is difficult to determine the biological significance of the detected low-magnitude changes, it could be that our method is identifying shifts between epigenetic marks in a particular cell type. The data sourced for this pilot project were not cell type-specific, so we may see a magnified effect of disease status at the identified DIPs in sorted or isolated cell populations. These considerations highlight the additional biological information that our proposed statistical approach provides compared with a traditional analysis and emphasize the importance of applying this approach to specific cell populations.

While our results suggest that our interaction model has increased sensitivity compared with separate models, there are still some limitations to this new method. First, interaction terms complicate interpretation of the coefficients for other terms in the model, making it difficult to quickly understand how disease is affecting 5-mC/5-hmC beta values. Second, interaction terms require a larger sample size than normal fixed effect terms, which means this approach may not be appropriate for smaller datasets. Third, the biological significance of subtle shifts in 5-mC and 5-hmC trajectories by disease status remains to be seen and requires further study. However, given the existence of unique “readers” and “writers” for each mark, it is plausible that subtle changes could have profound effects on gene expression and cellular function (Cheng et al., 2015). Finally, the interaction term model that we used only provides information on how the balance between 5-mC and 5-hmC shifts with AD, not the main effect of disease on each individual epigenetic mark. To understand how AD affects either 5-mC or 5-hmC on their own, it would be more appropriate to analyze the marks as independent data (see section 4). Despite these limitations, the analysis described in this paper may be useful for researchers interested in understanding how disease affects the interplay between 5-mC and 5-hmC in the brain.

## Conclusions and Next Steps

Recent research has developed a number of methods for measuring genome-wide 5-hmC. These methods continue to improve and provide exciting new opportunities for understanding the biological role of DNA modifications. However, despite an abundance of available technical methods, it remains unclear how to best reconcile paired, base-pair resolution 5-mC and 5-hmC data. Here, we proposed a statistical approach to handle 5-mC and 5-hmC as repeated measures using ME models with an interaction term between experimental condition and DNA modification category. As a proof of concept, we piloted this method using publicly available data. In our pilot analysis, we showed that the proposed statistical method would allow for a more complete understanding of the interplay between 5-mC and 5-hmC in nervous system tissue, a necessary step on the road to designing targeted epigenetic therapeutics for neurological diseases. Moving forward, the proposed statistical approach should be further verified in datasets with much larger sample sizes. In addition, future studies should test goodness of fit for the proposed interaction term modeling approach on these larger cohorts using established statistical tests. Finally, the changes in 5-mC and 5-hmC identified using the proposed method should be verified using targeted assays such as pyrosequencing. Using larger sample sizes and verifying specific CpGs will help to resolve the question discussed earlier concerning the appropriate FDR cutoff in this type of analysis. With these additional verifications, we hope that this method will allow researchers to better understand the interplay of 5-mC and 5-hmC in the brain.

## Data Availability

Publicly available datasets were analyzed in this study. This data can be found here: https://www.ncbi.nlm.nih.gov/geo/query/acc.cgi?acc=GSE105109.

## Author Contributions

JK, CS, and AB contributed to the conception and development of the hypothesis. JK developed the statistical model. CS wrote the first draft of the manuscript. JK wrote the subsequent drafts. All authors contributed to manuscript revision and read and approved the submitted version.

## Funding

This work was supported by NIEHS R00ES024570 and R21ES029205.

## Conflict of Interest Statement

The authors declare that the research was conducted in the absence of any commercial or financial relationships that could be construed as a potential conflict of interest.

## Supplementary Material

Supplementary Material List of significant differential probes, including those from interaction term modeling (5mC_5hmC_interaction_probes.csv), those from separate models for each epigenetic mark (5mC_probes.csv, 5hmC_probes.csv), and those probes that overlapped between the separate models (5mC_5hmC_overlap_probes.csv). The code used to perform all analyses is provided as an Rmarkdown file (BS_oxBS_Analysis_Kochmanski.Rmd) and HTML file (BS_oxBS_Analysis_Kochmanski.html). Quality control figures from both control probe tests and ChAMP are provided in labeled folders (ChAMP_Raw, ChAMP_ssNoob, CHAMP_SVD_BS, CHAMP_SVD_OX, and Control_Probes).
