# Supplementary material for: A Novel Application of Mixed Effects Models for Reconciling Base-Pair Resolution 5-Methylcytosine and 5-Hydroxymethylcytosine Data in Neuroepigenetics
Source: Front Genet. 2019 Sep 10;10:801. doi: 10.3389/fgene.2019.00801 (PMC6748167; doi:10.3389/fgene.2019.00801)
Supplement: Supplementary Material — List of significant differential probes, including those from interaction term modeling (5mC_5hmC_interaction_probes.csv), those from separate models for each epigenetic mark (5mC_probes.csv, 5hmC_probes.csv), and those probes that overlapped between the separate models (5mC_5hmC_overlap_probes.csv). The code used to perform all analyses is provided as an Rmarkdown file (BS_oxBS_Analysis_Kochmanski.Rmd) and HTML file (BS_oxBS_Analysis_Kochmanski.html). Quality control figures from both control probe tests and ChAMP are provided in labeled folders (ChAMP_Raw, ChAMP_ssNoob, CHAMP_SVD_BS, CHAMP_SVD_OX, and Control_Probes). [file Presentation_1.zip › Output copy/BS_oxBS_Analysis_Kochmanski.html]

BS/oxBS Array Analysis


Code 

- Show All Code
- Hide All Code

# BS/oxBS Array Analysis

#### 7/10/2019

## Project Summary

This script analyzes paired BS/oxBS data from the Illumina Methylation450K array and returns differentially modified cytosines.

1. Import the data in using minfi and set the annotation to the latest version of the 450k hg19 annotations (ilmn12.hg19) or EPIC array annotations (ilm10b4.hg19).
2. Identify probe-level detection p-values.
3. Filter probes that have a detection p-value > 0.01 in >10% of samples and probes that failed in any sample.
4. Mask cross-reactive and SNP probes based on Chen et al. 2013 (PMID:23314698) for 450K or Zhou et al. 2017 (PMID:27924034) for EPIC. Files from Chen et al. can be found here: http://www.sickkids.ca/Research/Weksberg-Lab/Publications/index.html. Documentation and download for Zhou et al. can be found here: http://zwdzwd.github.io/InfiniumAnnotation.
5. Dye bias correction with ssNoob in minfi.
6. Estimate cell type proportions with CETS.
7. Check for batch effects in ChAMP.
8. Estimate 5-mC and 5-hmC beta values with oxBS.MLE in ENmix.
9. Filter probes with mean 5-mC or 5-hmC beta value < 0.1 using dplyr. This removes issue of zero-inflation.
10. Remove any probes with missing 5-mC or 5-hmC beta values.
11. Test for differentially methylated AND hydroxymethylated CpGs using paired gamlss model with interaction term.
12. Test for differentially methylated or hydroxymethylated CpGs using separate gamlss models.
13. Compare outputs from separate models and paired model.

## Data setup

This assumes you have already downloaded the data and prepped a sample sheet, but you can add a step to download the data directly from GEO, and create the sample sheet as needed. The sample sheet must include unique sample names in the first column, a column for Assay where 1 indicates BS and 2 indicates oxBS, a column with Subject name such that there are 2 samples/assays per subject, all relevant data about the samples, and all data from running the arrays, including basename relative to this Rmd document. CETS must be download from Dr. Zachary Kaminsky and the appropriate data for probe masking must also be downloaded from the relevant publication.

- Parent Directory
  - This Rmd document
  - meta\_data.csv (file containing phenotypic data and sample sheet data)
  - idat (folder contains iScan output files)
  - CETS\_3.03 (folder contains CETS package, available from Dr. Zachary Kaminsky)
  - Manifest (folder contains manifest and/or files from Chen et al. 2013 (PMID:23314698) or Zhou et al. 2017 (PMID:27924034))
  - Output (empty folder for output)
    - ChAMP\_Raw (empty folder for ChAMP QC output on raw data)
    - ChAMP\_ssNoob (empty folder for ChAMP QC output on normalized data)
    - Control\_Probes (empty folder for control probe graphs)
  - Illumina manifest file for 450K or EPIC is csv format (download available at: https://support.illumina.com/downloads/infinium\_humanmethylation450\_product\_files.html or https://support.illumina.com/downloads/infinium-methylationepic-v1-0-product-files.html)

## 1. Load required packages

Load required packages.

```
library(minfi)
library(ChAMP)
library(ENmix)
library(tidyr)
library(dplyr)
library(plyr)
library(gamlss)
library(parallel)
```

## 2. Important idat files and set annotation

Set annotation to EPIC or 450K as appropriate.

```
# Import meta data
meta <- read.csv("./meta_data.csv", header = TRUE)

# Create RGChannelSet objects
rgset <- read.metharray.exp(targets = meta, recursive = TRUE, verbose = TRUE, force = TRUE, extended = TRUE)

# Set annotation
rgset@annotation = c(array='IlluminaHumanMethylation450k', annotation='ilmn12.hg19')
#rgset@annotation = c(array='IlluminaHumanMethylationEPIC', annotation='ilm10b4.hg19')
```

## 3. Check control probes

Check internal control probes with the ENmix function `plotCtrl`.  
Plots are saved in directory ./Output/Control Probes.

```
setwd("./Output/Control_Probes")
plotCtrl(rgset)
```

## 4. Generate MethylSet

Generate MethylSet without normalization using the minfi function `preprocessRaw`.

```
# Set sample names
sampleNames(rgset) <- rgset[[1]]

#Pull out phenotype data
pd <- pData(rgset)

# Set slide column to factor to avoid issues with champ.SVD
pd$Slide <- as.factor(pd$Slide) 

# Generate a MethylSet
mset <- preprocessRaw(rgset)

# Extract the beta values
raw_betas <- getBeta(mset, "Illumina")
```

## 5. Filter probes and samples

Generate detection p-values and filter probes with detP < 0.01. Remove probes that failed in > 5% of samples and samples with > 10% probes missing. User can set cutoffs for probe and sample filtering.

```
# Extract detection p-values
detP <- detectionP(rgset)

# Filter low quality probes based on detection value
raw_betas[detP >= 0.01] <- NA

# Calculate the proportion of probes that failed the detection p-value threshold
numfail <- matrix(colMeans(is.na(raw_betas)))

# Rename rows and columns
rownames(numfail) <- colnames(detP)
colnames(numfail) <- "Failed CpG Fraction"

# Identify samples with greater than 10% of probes failed
RemainSample <- which(numfail < 0.1)
RemoveSample <- which(numfail > 0.1)
RemoveSamples <- rownames(numfail)[RemoveSample]                     
RemoveSubject <- meta$subject[meta$X == RemoveSamples]
meta.r <- meta[RemainSample,]
RemainPairs <- meta.r$X[meta.r$subject != RemoveSubject]

# Remove samples with greater than 10% of failes probes and the corresponding paired sample
mset.r <- mset[,RemainPairs]
raw_betas.r <- raw_betas[,RemainPairs]
detP.r <- detP[,RemainPairs]

meta.f <- meta[RemainPairs,]
rgset.f <- rgset[, as.integer(RemainPairs)]
pd.f <- pd[RemainPairs,]

# Set the probe cutoff to drop probes that failed in > 5% of samples 
ProbeCutoff <- 0.05

# Remove probes that failed in > 5% of samples
mset.f <- mset.r[rowSums(is.na(raw_betas.r)) <= ProbeCutoff * ncol(detP), ]
raw_betas.f <- raw_betas.r[rowSums(is.na(raw_betas.r)) <= ProbeCutoff * ncol(detP), ]
detP.f <- detP.r[rowSums(is.na(raw_betas.r)) <= ProbeCutoff * ncol(detP),]
```

70 of 72 samples remain after filtering samples with a high level (>10%) of failed probes and any corresponding paired samples.  
Of 485512 probes, 464137 remain after removing probes that failed in > 5% of samples.  
21375 probes were removed.

## 6. Mask probes

Select probe-lists from Chen for 450k or from Zhou for EPIC.

```
# Chen (for 450k)
# Load files and filter for SNPs with MAF > 0.05
cross.react <- read.csv('./Manifest/48639-non-specific-probes-Illumina450k.csv', head = T, as.is = T)
cross.react.probes <- as.character(cross.react$TargetID)

commonSNP = read.table("./Manifest/48640-polymorphic-CpGs-Illumina450k_CpG.txt", header = TRUE,fill=TRUE)
commonSNP.f <- commonSNP[commonSNP$AF > 0.05, ]

SNP.SBE = read.table("./Manifest/48640-polymorphic-CpGs-Illumina450k_SBE.txt", header = TRUE,fill=TRUE)
SNP.SBE.f <- SNP.SBE[SNP.SBE$AF > 0.05, ]

# Combine into one object and remove duplicate probes
maskname = c(as.vector(cross.react$TargetID),as.vector(commonSNP.f$PROBE),as.vector(SNP.SBE.f$PROBE))
maskname = unique(maskname) 

# Filter mset and raw_betas to remove probes
mset.fm <- mset.f[!featureNames(mset.f) %in% maskname, ]
raw_betas.fm <- raw_betas.f[!rownames(raw_betas.f) %in% maskname, ]
detP.fm <- detP.f[!rownames(detP.f) %in% maskname, ]

# Zhou (for EPIC)
# Load files
#load("./Manifest/EPIC.manifest.rda")
#EPIC_manifest_hg19 <- as.data.frame(EPIC.manifest)
# Select probes to mask (see documentation)
#maskname <- rownames(EPIC_manifest_hg19)[which(EPIC_manifest_hg19$MASK.general == TRUE)]

# Filter mset and raw_betas to remove probes
#mset.fm <- mset.f[!featureNames(mset.f) %in% maskname, ]
#raw_betas.fm <- raw_betas.f[!rownames(raw_betas.f) %in% maskname, ]
```

After masking, 386245 probes remain. 77892 probes were removed.

## 7. Adjust beta-values

Fix Beta values that are either 0 or greater than or equal to 1.

```
if (min(raw_betas.fm, na.rm = TRUE) <= 0)
  raw_betas.fm[raw_betas.fm <= 0] <- min(raw_betas.fm[raw_betas.fm > 0])

if (max(raw_betas.fm, na.rm = TRUE) >= 1)
  raw_betas.m.fm[raw_betas.fm >= 1] <- max(raw_betas.fm[raw_betas.fm < 1])
```

Zeros in your dataset have been replaced with smallest positive value.  
Ones in your dataset have been replaced with largest value below 1.

## 8. Extract raw data and run QC

```
#Get the intensity values and detection p-values using minfi to feed to QC in ChAMP
intensity <- minfi::getMeth(mset.fm) + minfi::getUnmeth(mset.fm)
#detP <- detP.f[which(row.names(detP) %in% row.names(raw_betas.fm)), ]

#Compile the data into a list object to feed into ChAMP QC
preprocessed.raw.data <- list(mset = mset.fm, rgSet = rgset.f, pd = pd.f, intensity = intensity, beta = raw_betas.fm, detP = detP.fm)

#Run a QC with ChAMP
champ.QC(beta = preprocessed.raw.data$beta, pheno = pd.f$post.mortem.diagnosis.ch1, resultsDir = "./Output/ChAMP_Raw")
```

Plots generated by ChampQC can be found in ./Output/ChAMP\_Raw

## 9. Dye bias correction with ssNoob and run QC

Perform dye bias correction for beta values with ssNoob on probes remaining after filtering and masking. We do not need to separate BS and oxBS at this step since we are using single sample Noob.

```
# Perform dye bias correction for beta values with ssNoob on probes remaining after filtering and masking. We can leave BS and oxBS together because this is single sample Noob.
mset.n <- preprocessNoob(rgset.f, dyeMethod = "single")[rownames(raw_betas.fm), ]

# Set sample names
sampleNames(mset.n) = mset.n[[1]]

# Extract data from mset
betas.n <- getBeta(mset.n, "Illumina")

#Compile the data into a list object to feed into ChAMP QC
ssNoob.data <- list(mset = mset.n, rgSet = rgset.f, pd = pd.f, intensity = intensity, beta = betas.n, detP = detP.fm)

# Run QC in ChAMP after normalization
champ.QC(beta = ssNoob.data$beta, pheno = pd.f$post.mortem.diagnosis.ch1, resultsDir = "./Output/ChAMP_ssNoob/")
```

Plots generated by ChampQC can be found in ./Output/ChAMP\_ssNoob

## 10. Estimate cell type proportions with CETS

CETS (cell epigenotype specific) estimates neuronal and glial proportions based on methylation data (BS only). User should select appropriate controls for CETS.

```
# Select beta values for BS assays only in the remaining samples after filtering
bs <- as.character(meta.f$X[which(meta.f$Assay == 1)])
betas.n.bs <- betas.n[,bs]

#Load CETS
load("./CETS_3.03/CETS_Image.RData")

# Use glial cell proportion, control brains, Caucasian samples and male samples to generate reference 
idx <- list(controlNeuron = pdBrain$celltype == "N" & pdBrain$diag == "Control" & pdBrain$ethnicity == "Caucasian" & pdBrain$sex == "Male", controlGlia = pdBrain$celltype == "G" & pdBrain$diag == "Control" & pdBrain$ethnicity == "Caucasian" & pdBrain$sex == "Male")
refProfile <- getReference(brain, idx)

# Estimate neuronal proportion
prop.n <- estProportion(betas.n.bs, profile = refProfile)
round(prop.n, 3)

# Convert to glial proportion (1- neuronal proportion)
prop.g <- as.data.frame(1 - prop.n) 

# Change rowname to column
prop.g <- tibble::rownames_to_column(prop.g, var = "rowname")

# Change column name
names(prop.g)[1] <- "Sample"

# Change column name to glial
names(prop.g)[2] <- "glial"

# Keep case and glial cell proportion
keep <- c("Sample", "glial")
prop.g <- prop.g[,keep]
prop.g$subject <- NA
prop.g$subject <- meta.f$subject[match(prop.g$Sample, meta.f$X)]

# Add glial cell proportion data to meta data
meta.f$glial <- NA 
meta.f$glial <- prop.g$glial[match(meta.f$subject, prop.g$subject)]

# Add glial cell proportion data to pd
pd.f@listData$glial <- NA
pd.f@listData$glial <- prop.g$glial[match(pd.f@listData$subject, prop.g$subject)]
```

```
## GSM2818020 GSM2818026 GSM2818028 GSM2818030 GSM2818040 GSM2818048 
##      0.000      0.366      0.449      0.128      0.139      0.154 
## GSM2818052 GSM2818060 GSM2818062 GSM2818064 GSM2818070 GSM2818074 
##      0.447      0.098      0.298      0.096      0.000      0.317 
## GSM2818076 GSM2818080 GSM2818084 GSM2818086 GSM2818092 GSM2818096 
##      0.354      0.426      0.160      0.315      0.406      0.219 
## GSM2818100 GSM2818102 GSM2818106 GSM2818120 GSM2818122 GSM2818124 
##      0.175      0.204      0.458      0.109      0.190      0.371 
## GSM2818144 GSM2818146 GSM2818148 GSM2818156 GSM2818160 GSM2818166 
##      0.325      0.105      0.097      0.220      0.371      0.198 
## GSM2818170 GSM2818172 GSM2818186 GSM2818204 GSM2818208 
##      0.306      0.302      0.174      0.466      0.422
```

## 11. Separate data by assay

```
ox <- as.character(meta.f$X[which(meta.f$Assay == 2)])
betas.n.ox <- betas.n[,ox]

intensity.bs <- intensity[,bs]
intensity.ox <- intensity[,ox]

pd.bs <- pd.f[which(pd.f$Assay == "1"),]
pd.ox <- pd.f[which(pd.f$Assay == "2"),]
```

## 12. Run ChAMP SVD

Run SVD to determine which variables to include in downstream modeling.

```
champ.SVD(beta = betas.n.bs, pd = pd.bs, resultsDir = "./Output/CHAMP_SVD_BS/")
```

```
champ.SVD(beta = betas.n.ox, pd = pd.ox, resultsDir = "./Output/CHAMP_SVD_OX/")
```

SVD Summary files can be found in ./Output/CHAMP\_SVD\_BS and ./Output/CHAMP\_SVD\_OC

## 13. oxBS.MLE

oxBS.MLE requires colnames to match for beta and intensity value objects. This section will depend on how your samples and subjects are named, but is simple if you created a subject ID assigned to each pair in your sample sheet/meta data.

```
# Set column names to be the same
colnames(betas.n.bs) <- pd.bs@listData$subject[colnames(betas.n.bs) == pd.bs@listData$X]
colnames(betas.n.ox) <- pd.ox@listData$subject[colnames(betas.n.ox) == pd.ox@listData$X]

colnames(intensity.bs) <- pd.bs@listData$subject[colnames(intensity.bs) == pd.bs@listData$X]
colnames(intensity.ox) <- pd.ox@listData$subject[colnames(intensity.ox) == pd.ox@listData$X]
```

```
MLE <- oxBS.MLE(beta.BS = betas.n.bs, beta.oxBS = betas.n.ox, N.BS = intensity.bs, N.oxBS = intensity.ox)

# Combine  matrices into dataframe
mc <- as.data.frame(MLE$`5mC`)
hmc <- as.data.frame(MLE$`5hmC`)
```

5-mC and 5-hmC beta values have been estimated.

```
#Save estimated 5-mC and 5-hmC beta values
save(mc, hmc, file = "./Output/oxBS-MLE_betas.RData")
```

oxBS.MLE output has been saved as an RData file in the Output directory.

## 14. Prep data for modeling

Filter probes with beta value < 0.1 for 5-hmC and/or 5-mC prior to modeling.

```
# Load estimated 5-mC and 5-hmC beta values for oxBS-450K data if needed
#load("./Output/oxBS-MLE_betas.RData")

# Create columns of means
mc$mean_5mC <- rowMeans(mc, na.rm=TRUE)
hmc$mean_5hmC <- rowMeans(hmc, na.rm=TRUE)

# Establish probe variable using rownames
mc$probe <- rownames(mc)
hmc$probe <- rownames(hmc)

# Filter 5-hmC and 5-mC data to remove probes with mean beta value < 0.10
mc.f <- filter(mc, mean_5mC > 0.1)
hmc.f <- filter(hmc, mean_5hmC > 0.1)
```

Prep data for downstream modeling. This includes and optional step to chunk the data for multiple node processing. The size and number of these chunks will depend on number of probes and nodes available.

```
# Merge 5-mC and 5-hmC data for remaining probes and transpose dataframe for downstream processing
bvals <- plyr::join(mc.f,hmc.f, by = "probe", type = "inner") 
row.names(bvals) <- bvals$probe 

# Remove extra columns
bvals$probe <- NULL
bvals$mean_5mC <- NULL
bvals$mean_5hmC <- NULL
bvals.complete <- bvals[complete.cases(bvals),]

# Transpose for regression
bvals.t <- as.data.frame(t(bvals.complete))

# Adjust beta values
if (min(bvals.t, na.rm = TRUE) <= 0)
  bvals.t[bvals.t <= 0] <- min(bvals.t[bvals.t > 0])

if (max(bvals.t, na.rm = TRUE) >= 1)
  bvals.t[bvals.t >= 1] <- max(bvals.t[bvals.t < 1])

# Optional: Test on a small number of probes
#bvals.t <- bvals.t[,c(1:500)]

# Optional: Chunk the data for multiple node processing if necessary
#bvals.t.chnk1 <- bvals.t[,c(1:50000)]
#bvals.t.chnk2 <- bvals.t[,c(50001:100000)]
#bvals.t.chnk3 <- bvals.t[,c(100001:150353)]
```

After filtering, 146773 probes were removed from mC beta values and 231356 probes were removed from hmC beta values. 70 probes remain with data for both mC and hmC for downstream modeling.

## 15. Interaction modeling

Create covariates from the metadata for downstream modeling. These must be set by the user based on the research question. Here we include only glial cell proportion and age. We did not include sex since we have preselected only males samples.

```
# Set up appropriate covariates
glial <- meta.f$glial
age <- meta.f$age.at.death.ch1

# Create predictor variables from metadata for downstream modeling; these will vary based on the research question.
disease <- meta.f$post.mortem.diagnosis.ch1
DNA_mod_cat <- meta.f$Assay 

# Length of all variables should be the same as the number of sample IDs:

# Build paired ID as a random effect (Assay = A or B, depending on 5-mC or 5-hmC)
pairedID <- nrow(meta.f)/2
randeff <- as.factor(c(1:pairedID,1:pairedID))
```

### gamlss beta regression modeling for paired 5-mC and 5-hmC data using interaction term.

User will need to specify which variable corresponds to the interaction term, as this may change based on how many covariates are included in the model.

```
#Establish function for mixed effects beta regression with a logit link function in gamlss R package:
Ilm450k.beta.fit.full <- function(x, glial, age, randeff) {
  x <- as.data.frame(x)
  x$randeff <- randeff
  x$glial <- glial
  x$age <- age

# Define data; this will vary depending on sample size of study.
#data <- x[1:70,]
data <- x[1:nrow(meta.f),]

# Fit models
none.fit <- gamlss(x ~ disease*DNA_mod_cat + glial + age, random = ~1 | randeff, data = data, family = BE, trace = F)
  
# Extract the estimate, se, t-value and p-value for beta coefficient (interaction term)
none.fit.sum <- as.data.frame(summary(none.fit))[6,c(1:4)] 
  #Note: May need to change the [6,c(1:4)], depending on how many terms are in the model.
  
  return(none.fit.sum)
}

# Run the model
fit <- mclapply(as.data.frame(bvals.t), function(x) Ilm450k.beta.fit.full(x, glial, age, randeff), mc.preschedule = F, mc.cores = 4)
```

```
# This will need to be expanded if data was split into chunks above.

# Create data frames of modeling results and add variable name:
fit <- ldply(fit, data.frame)
```

Model has been run for 150353 probes.

Beta regression results for the first 3 probes.

c(“cg05230942”, “cg08921682”, “cg11131351”), c(0.541874318792512, -0.21244507593719, 1.66566235968476), c(0.400772167416, 0.539461578217066, 0.451752114647333), c(1.35207572493439, -0.393809465799818, 3.68711580018854), c(0.181185129019797, 0.695051463580513, 0.00047428333272143)

### Multiple testing correction using false discovery rate

User can set appropriate FDR cutoff and standard error cutoffs.

```
# Pull out p-values from modeling results data frame:
p1 <- fit$Pr...t..

# Adjust p-values using Benjamini-Hochberg FDR adjustment method in p.adjust function
fdr <- p.adjust(p1, method = "BH", n = length(p1)) 

# Append the FDR values to the dataset.
fit$fdr <- fdr

# Filter down to probes with standard error less than 10 to remove any probes with high standard errors. These would be poorly modeled in this analysis.
fit.se <- fit[(fit$Std..Error < 10), ] 

# Filter and sort by FDR value (e.g. FDR<0.05):
fit.se.fdr <- fit.se[(fit.se$fdr < 0.05), ]
fit.se.fdr.sort <- fit.se.fdr[order(fit.se.fdr$fdr),]
```

After filtering for probes by standard error, 150353 of 150353 probes remain.

After filtering for probes by FDR, 1 remain.

### Annotate differential probe IDs

```
# Read in manifest (specify correct file for 450K or EPIC), remove headers and label probe ID as "probe"
Illumina_manifest <- read.csv("HumanMethylation450_15017482_v1-2.csv", skip = 7)
colnames(Illumina_manifest)[colnames(Illumina_manifest) == "Name"]<- "probe"

# Rename probe ID as "probe" in modeling output
colnames(fit.se.fdr.sort)[colnames(fit.se.fdr.sort) == ".id"] <- "probe"

#Merge data frames of gamlss interaction term model outputs with the EPIC manifest by "probe" variable:
fit.merged <- merge(fit.se.fdr.sort,Illumina_manifest,by=c("probe"))
```

Significant probes have been annotated.

## 16. Separate modeling of 5-mC and 5-hmC

Create covariates from the metadata for downstream modeling. Some of these have already been created above, but are included here for completeness. These covariates must be set by the user based on the research question. Here we include only glial cell proportion and age. We did not include sex since we preselected only male samples.

```
# Set up appropriate covariates
glial <- meta.f$glial
age <- meta.f$age.at.death.ch1

# Create predictor variables from metadata for downstream modeling; these will vary based on the research question.
disease <- meta.f$post.mortem.diagnosis.ch1
disease.mc <- disease[1:(length(disease)/2)]
disease.hmc <- disease[((length(disease)/2)+1):length(disease)]
```

### gamlss beta regression modeling for seperate 5-mC and 5-hmC datasets

User will need to specify which variable corresponds to the interaction term, as this may change based on how many covariates are included in the model.

```
# Establish function for separate beta regression models with logit link functions in gamlss R package:
Ilm450k.beta.fit.sep <- function(x, glial, age) {
  x <- as.data.frame(x)
  x$glial <- glial
  x$age <- age

  # Define data
  data.5mC <- x[1:(length(disease)/2),]
  data.5hmC <- x[((length(disease)/2)+1):length(disease),]
  
  # Fit the model for the 5-mC data
  mc.fit <- gamlss(x ~ disease.mc + glial + age, data = data.5mC, family = BE, trace = F)
  
  # Extract the estimate, se, t-value and p-value for beta coefficient (interaction term)
  mc.fit.sum <- as.data.frame(summary(mc.fit))[2,c(1:4)] 
  # Note: May need to change the [2,c(1:4)], depending on order of terms are in the model
  
  # Fit the model for the 5-hmC data
  hmc.fit <- gamlss(x ~ disease.hmc + glial + age, data = data.5hmC, family = BE, trace = F)
  
  # Extract the estimate, se, t-value and p-value for beta coefficient (interaction term)
  hmc.fit.sum <- as.data.frame(summary(hmc.fit))[2,c(1:4)] 
  
  return(list(model.5mC = mc.fit.sum,
              model.5hmC = hmc.fit.sum))
}

# Run the model
s.fit <- mclapply(as.data.frame(bvals.t), function(x) Ilm450k.beta.fit.sep(x, glial, age), mc.preschedule = F, mc.cores = 4)
```

```
# This will need to be expanded if there are more than 3 chunks of data above.

# Create data frames of modeling results and add variable name:
s.fit <- ldply (s.fit, data.frame)
```

Model has been run for 150353 probes.

Beta regression results for the first 3 probes.

c(“cg05230942”, “cg08921682”, “cg11131351”), c(-0.0779616495496538, 0.0126116356414684, -0.011988268303681), c(0.0745621878658402, 0.123055643360655, 0.115955451676672), c(-1.04559230061664, 0.102487259397814, -0.103386844950669), c(0.30383419283198, 0.919030022980061, 0.918321904328618), c(0.641206460961039, 0.17559632214945, 0.117184957877145), c(0.311210436045814, 0.343735664321831, 0.348229590173145), c(2.06036297853021, 0.510846968689998, 0.336516370762402), c(0.0478411748457875, 0.613077260361459, 0.738748976519836)

### Multiple testing correction using false discovery rate

User can set appropriate FDR cutoff and standard error cutoffs.

```
# Pull out p-values from 5-mC and 5-hmC modeling
p.mc <- s.fit$model.5mC.Pr...t..
p.hmc <- s.fit$model.5hmC.Pr...t..

# Adjust p-values using Benjamini-Hochberg FDR adjustment method in p.adjust function
fdr.mc <- p.adjust(p.mc, method = "BH", n = length(p.mc)) 
fdr.hmc <- p.adjust(p.hmc, method = "BH", n = length(p.hmc)) 

# Append the FDR values to the dataset.
s.fit$fdr.5mC <- fdr.mc
s.fit$fdr.5hmC <- fdr.hmc

# Filter down to probes with standard error less than 10 to remove any probes with high standard errors. These would be poorly modeled in this analysis.
s.fit.se <- s.fit[(s.fit$model.5mC.Std..Error < 10) & s.fit$model.5hmC.Std..Error < 10, ] 

# Filter and sort by FDR value (e.g. FDR<0.05):
s.fit.se.fdr.mc <- s.fit.se[(s.fit.se$fdr.5mC < 0.05), ]
s.fit.se.fdr.mc.sort <- s.fit.se.fdr.mc[order(s.fit.se.fdr.mc$fdr.5mC),] 

s.fit.se.fdr.hmc <- s.fit.se[(s.fit.se$fdr.5hmC < 0.05), ]
s.fit.se.fdr.hmc.sort <- s.fit.se.fdr.hmc[order(s.fit.se.fdr.hmc$fdr.5hmC),]
```

After filtering for probes by standard error, 150353 of 150353 probes remain.

After filtering for probes by FDR, there are 0 probes significant for 5mC and 1 probes significant for 5hmC.

### Annotate differential probe IDs

```
# Manifest was previously loaded

# Rename probe ID as "probe" in modeling output
colnames(s.fit.se.fdr.mc.sort)[colnames(s.fit.se.fdr.mc.sort) == ".id"] <- "probe"
colnames(s.fit.se.fdr.hmc.sort)[colnames(s.fit.se.fdr.hmc.sort) == ".id"] <- "probe"

# Merge data frames of gamlss interaction term model outputs with the EPIC manifest by "probe" variable:
s.fit.mc.merged <- merge(s.fit.se.fdr.mc.sort,Illumina_manifest,by=c("probe"))
s.fit.hmc.merged <- merge(s.fit.se.fdr.hmc.sort,Illumina_manifest,by=c("probe"))
```

Significant probes have been annotated.

Lists of significant probes for each modeling scenario can now be explored and visualized as needed.

## 17. Examine the overlap of the two separate gamlss analyses.

```
# Extract probes where BOTH 5-mC and 5-hmC have FDR < 0.05:
s.fit.both <- s.fit.se[(s.fit.se$fdr.5mC < 0.05 & s.fit.se$fdr.5hmC < 0.05), ] 

# Rename column ".id" as "probe"
colnames(s.fit.both)[colnames(s.fit.both) ==".id"] <- "probe"
```

0 probes were significant in separate modeling of both 5-mC and 5-hmC.

## 18. Examine the overlap between interaction term modeling and separate modeling.

```
# Check for overlap between the interaction term models and the probe IDs that overlapped in separate models:
overlap <- merge(s.fit.both, fit.merged, by = "probe")
```

0 differentially modified probes were identified in both interaction modeling and in both marks by separate modeling.

## 19. Save output

```
save(fit.merged, s.fit.mc.merged, s.fit.hmc.merged, s.fit.both, overlap, file = "./Output/significant_probes.RData")
```
