# Supplementary figures and images for: A Novel Application of Mixed Effects Models for Reconciling Base-Pair Resolution 5-Methylcytosine and 5-Hydroxymethylcytosine Data in Neuroepigenetics
Source: Front Genet. 2019 Sep 10;10:801. doi: 10.3389/fgene.2019.00801 (PMC6748167; doi:10.3389/fgene.2019.00801)

## Density plot of raw data (386245 probes)

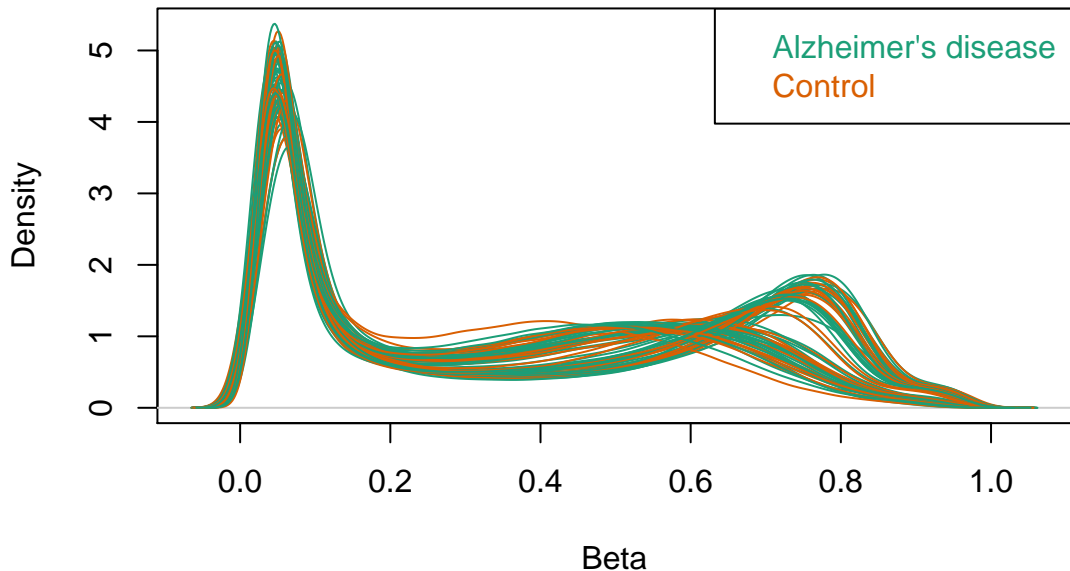

Supplement: Supplementary Material — List of significant differential probes, including those from interaction term modeling (5mC_5hmC_interaction_probes.csv), those from separate models for each epigenetic mark (5mC_probes.csv, 5hmC_probes.csv), and those probes that overlapped between the separate models (5mC_5hmC_overlap_probes.csv). The code used to perform all analyses is provided as an Rmarkdown file (BS_oxBS_Analysis_Kochmanski.Rmd) and HTML file (BS_oxBS_Analysis_Kochmanski.html). Quality control figures from both control probe tests and ChAMP are provided in labeled folders (ChAMP_Raw, ChAMP_ssNoob, CHAMP_SVD_BS, CHAMP_SVD_OX, and Control_Probes). [file Presentation_1.zip › Output copy/ChAMP_Raw/raw_densityPlot.pdf]

All samples before normalization (386245 probes)

120  
100  
80  
60  
40  
20  
0

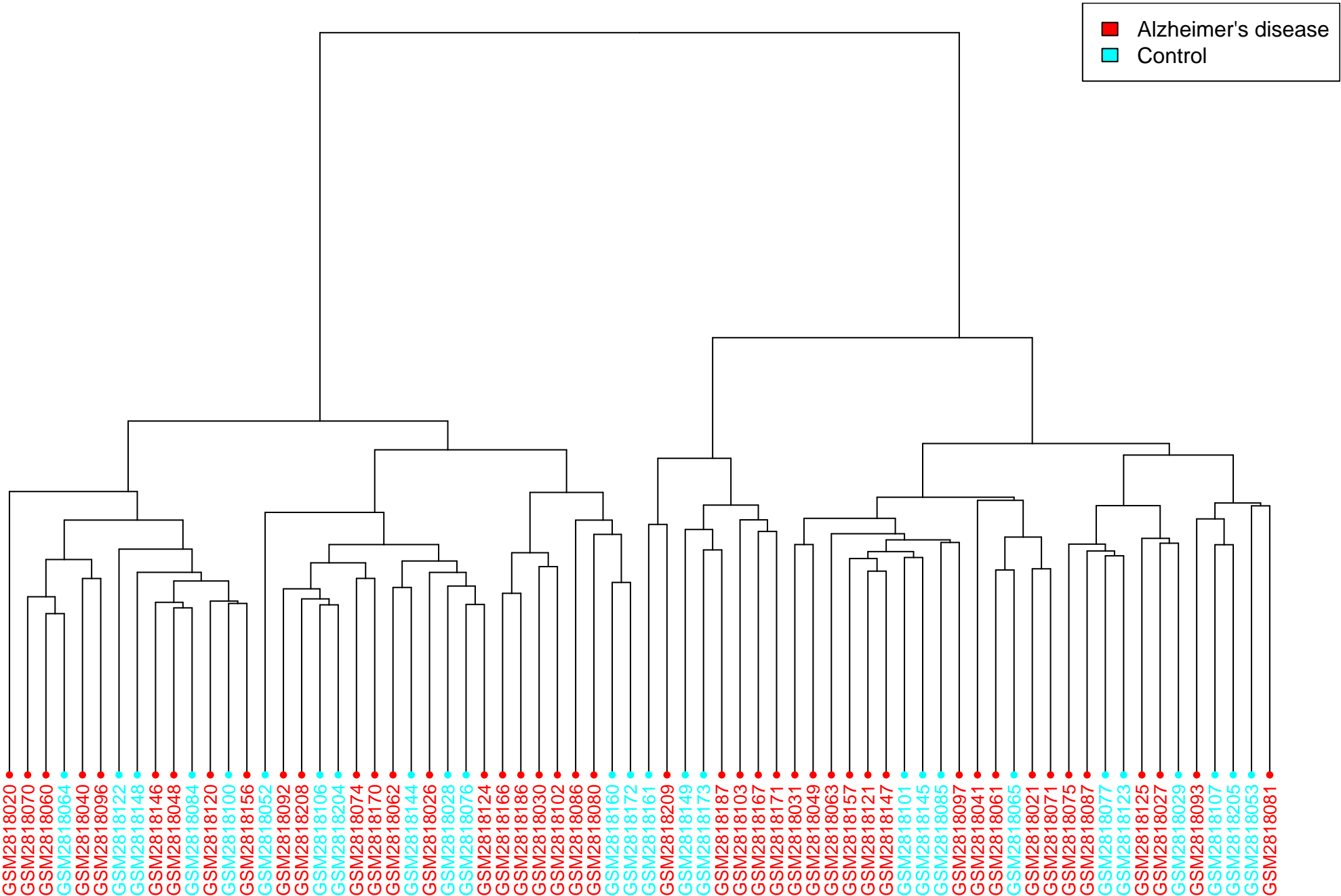

Supplement: Supplementary Material — List of significant differential probes, including those from interaction term modeling (5mC_5hmC_interaction_probes.csv), those from separate models for each epigenetic mark (5mC_probes.csv, 5hmC_probes.csv), and those probes that overlapped between the separate models (5mC_5hmC_overlap_probes.csv). The code used to perform all analyses is provided as an Rmarkdown file (BS_oxBS_Analysis_Kochmanski.Rmd) and HTML file (BS_oxBS_Analysis_Kochmanski.html). Quality control figures from both control probe tests and ChAMP are provided in labeled folders (ChAMP_Raw, ChAMP_ssNoob, CHAMP_SVD_BS, CHAMP_SVD_OX, and Control_Probes). [file Presentation_1.zip › Output copy/ChAMP_Raw/raw_SampleCluster.pdf]

## Density plot of raw data (386245 probes)

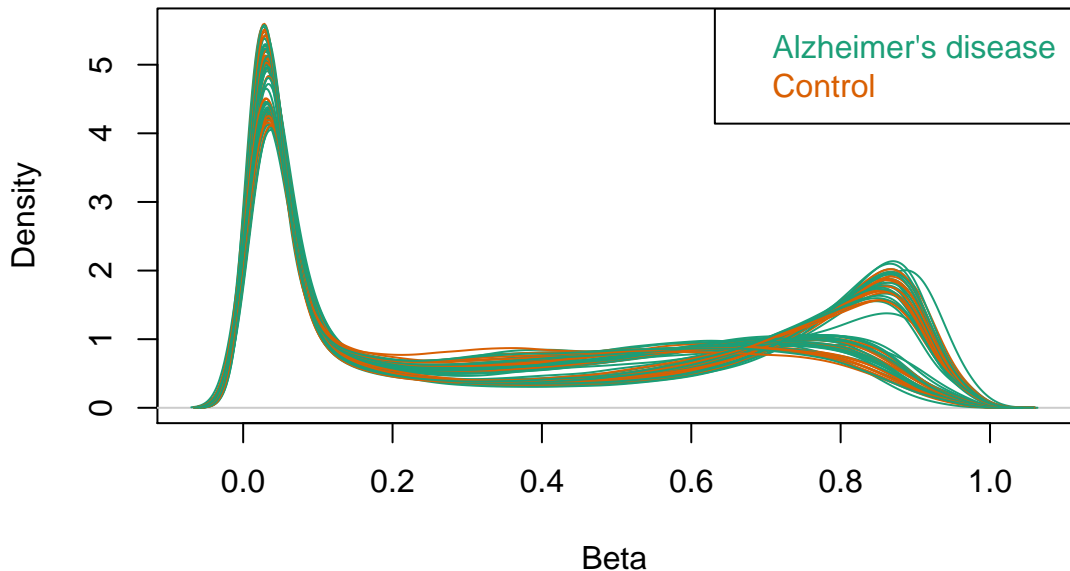

Supplement: Supplementary Material — List of significant differential probes, including those from interaction term modeling (5mC_5hmC_interaction_probes.csv), those from separate models for each epigenetic mark (5mC_probes.csv, 5hmC_probes.csv), and those probes that overlapped between the separate models (5mC_5hmC_overlap_probes.csv). The code used to perform all analyses is provided as an Rmarkdown file (BS_oxBS_Analysis_Kochmanski.Rmd) and HTML file (BS_oxBS_Analysis_Kochmanski.html). Quality control figures from both control probe tests and ChAMP are provided in labeled folders (ChAMP_Raw, ChAMP_ssNoob, CHAMP_SVD_BS, CHAMP_SVD_OX, and Control_Probes). [file Presentation_1.zip › Output copy/ChAMP_ssNoob/raw_densityPlot.pdf]

All samples before normalization (386245 probes)

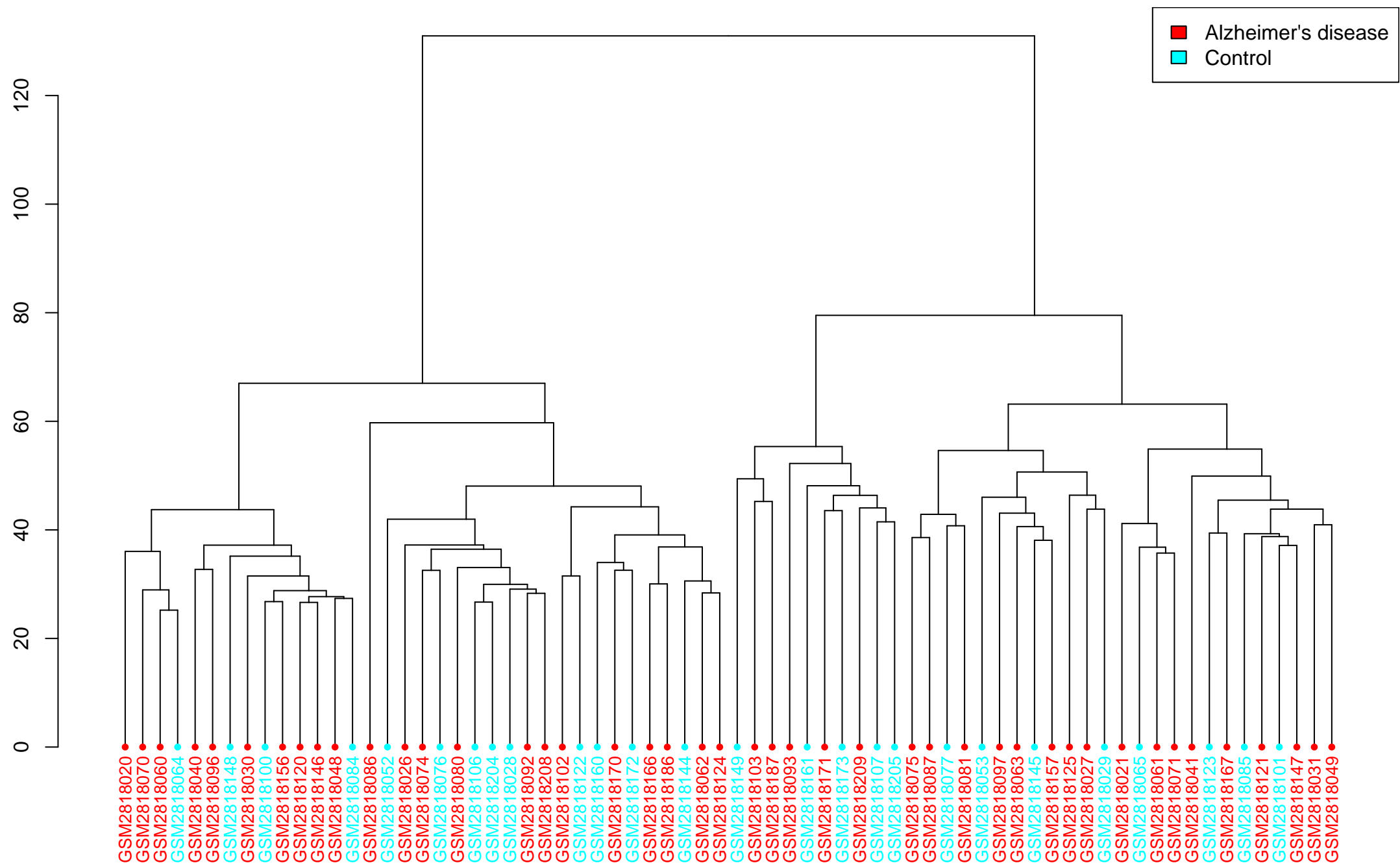

Supplement: Supplementary Material — List of significant differential probes, including those from interaction term modeling (5mC_5hmC_interaction_probes.csv), those from separate models for each epigenetic mark (5mC_probes.csv, 5hmC_probes.csv), and those probes that overlapped between the separate models (5mC_5hmC_overlap_probes.csv). The code used to perform all analyses is provided as an Rmarkdown file (BS_oxBS_Analysis_Kochmanski.Rmd) and HTML file (BS_oxBS_Analysis_Kochmanski.html). Quality control figures from both control probe tests and ChAMP are provided in labeled folders (ChAMP_Raw, ChAMP_ssNoob, CHAMP_SVD_BS, CHAMP_SVD_OX, and Control_Probes). [file Presentation_1.zip › Output copy/ChAMP_ssNoob/raw_SampleCluster.pdf]

# Singular Value Decomposition Analysis (SVD)

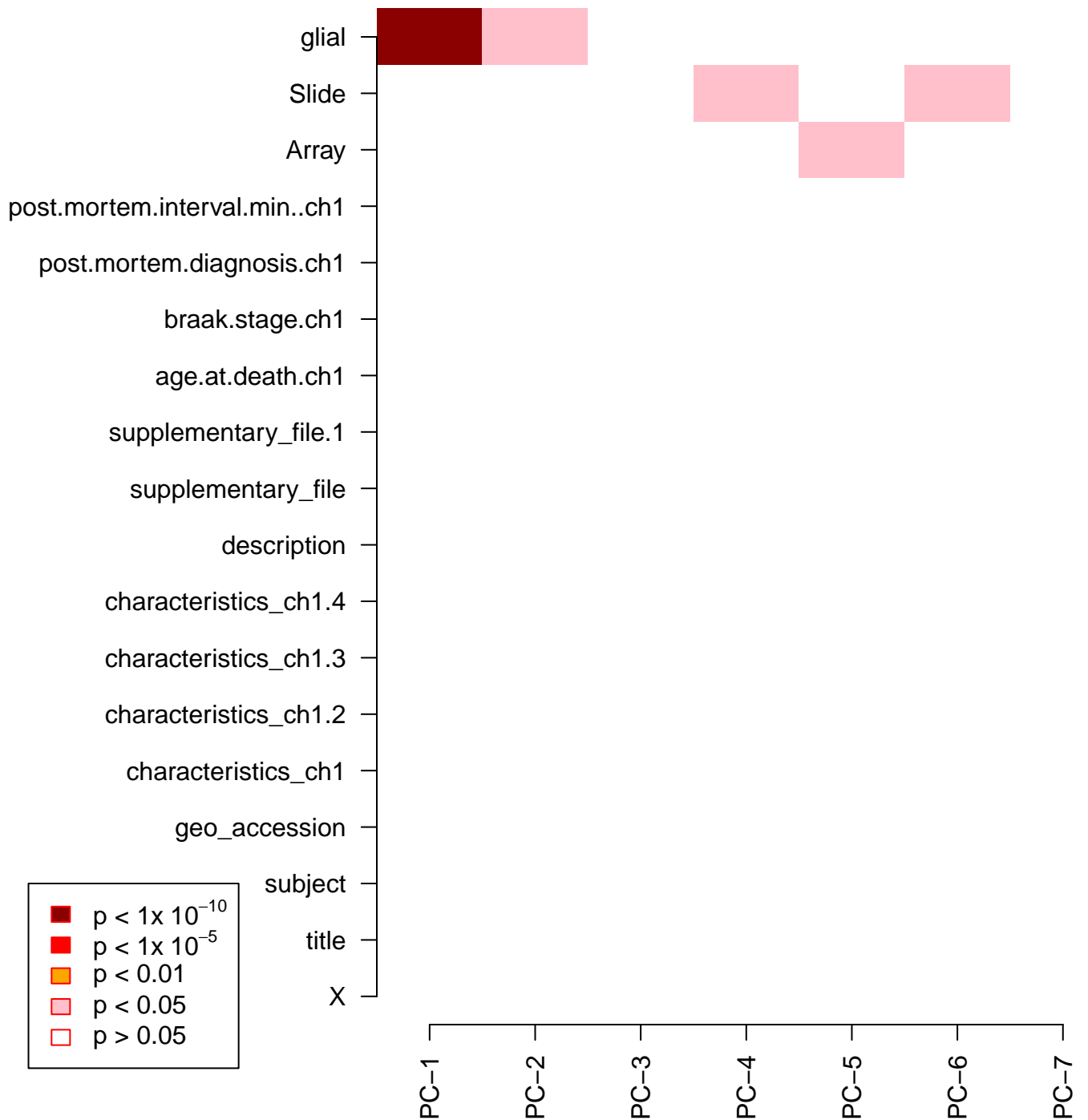

Supplement: Supplementary Material — List of significant differential probes, including those from interaction term modeling (5mC_5hmC_interaction_probes.csv), those from separate models for each epigenetic mark (5mC_probes.csv, 5hmC_probes.csv), and those probes that overlapped between the separate models (5mC_5hmC_overlap_probes.csv). The code used to perform all analyses is provided as an Rmarkdown file (BS_oxBS_Analysis_Kochmanski.Rmd) and HTML file (BS_oxBS_Analysis_Kochmanski.html). Quality control figures from both control probe tests and ChAMP are provided in labeled folders (ChAMP_Raw, ChAMP_ssNoob, CHAMP_SVD_BS, CHAMP_SVD_OX, and Control_Probes). [file Presentation_1.zip › Output copy/CHAMP_SVD_BS/SVDsummary.pdf]

# Singular Value Decomposition Analysis (SVD)

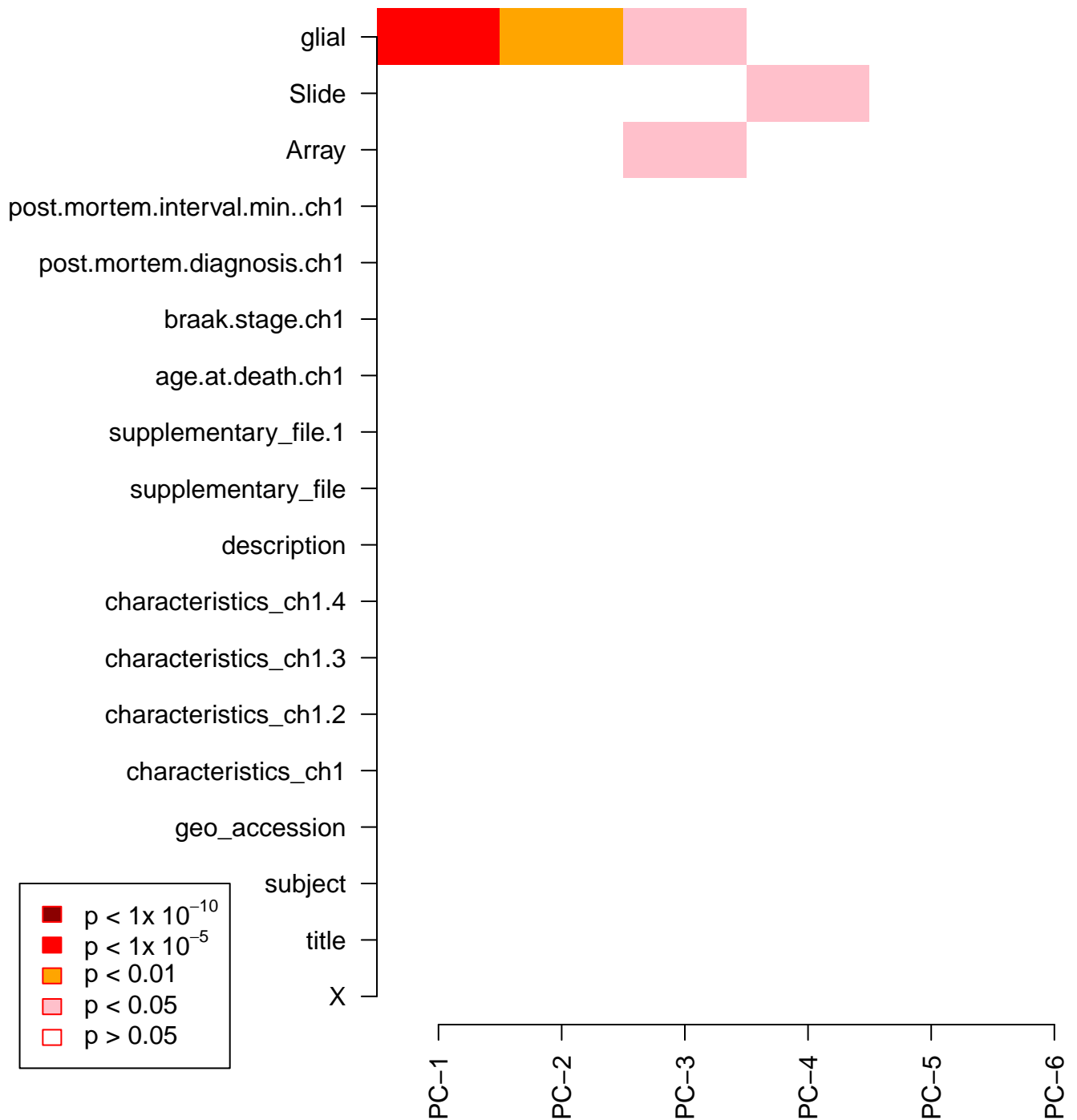

Supplement: Supplementary Material — List of significant differential probes, including those from interaction term modeling (5mC_5hmC_interaction_probes.csv), those from separate models for each epigenetic mark (5mC_probes.csv, 5hmC_probes.csv), and those probes that overlapped between the separate models (5mC_5hmC_overlap_probes.csv). The code used to perform all analyses is provided as an Rmarkdown file (BS_oxBS_Analysis_Kochmanski.Rmd) and HTML file (BS_oxBS_Analysis_Kochmanski.html). Quality control figures from both control probe tests and ChAMP are provided in labeled folders (ChAMP_Raw, ChAMP_ssNoob, CHAMP_SVD_BS, CHAMP_SVD_OX, and Control_Probes). [file Presentation_1.zip › Output copy/CHAMP_SVD_OX/SVDsummary.pdf]

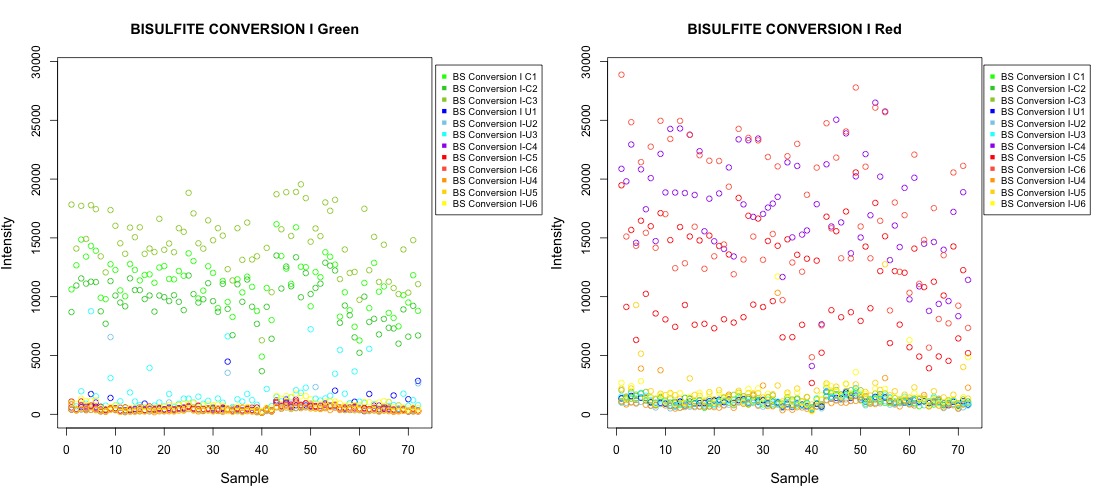

Supplement: Supplementary Material — List of significant differential probes, including those from interaction term modeling (5mC_5hmC_interaction_probes.csv), those from separate models for each epigenetic mark (5mC_probes.csv, 5hmC_probes.csv), and those probes that overlapped between the separate models (5mC_5hmC_overlap_probes.csv). The code used to perform all analyses is provided as an Rmarkdown file (BS_oxBS_Analysis_Kochmanski.Rmd) and HTML file (BS_oxBS_Analysis_Kochmanski.html). Quality control figures from both control probe tests and ChAMP are provided in labeled folders (ChAMP_Raw, ChAMP_ssNoob, CHAMP_SVD_BS, CHAMP_SVD_OX, and Control_Probes). [file Presentation_1.zip › Output copy/Control_Probes/BISULFITE_CONVERSION_I.jpg]

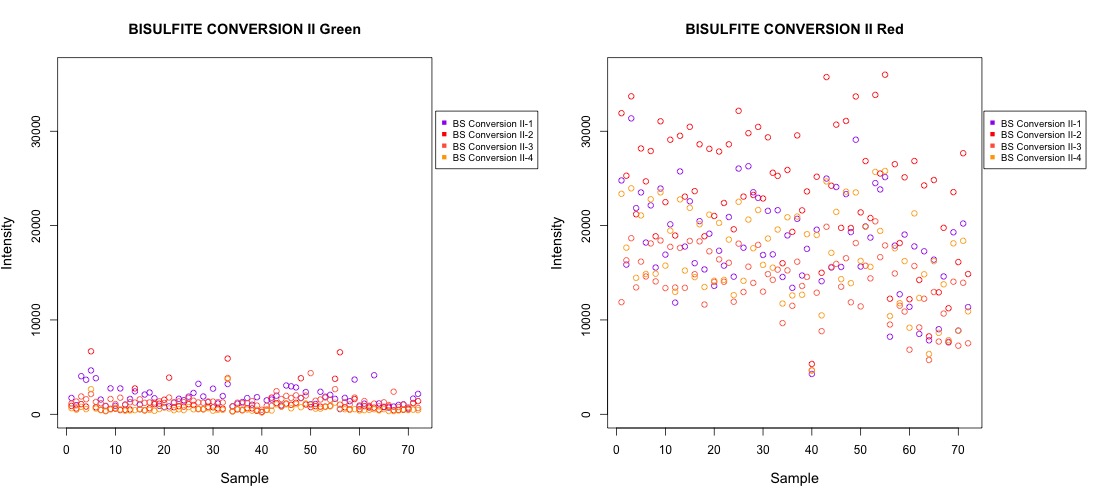

Supplement: Supplementary Material — List of significant differential probes, including those from interaction term modeling (5mC_5hmC_interaction_probes.csv), those from separate models for each epigenetic mark (5mC_probes.csv, 5hmC_probes.csv), and those probes that overlapped between the separate models (5mC_5hmC_overlap_probes.csv). The code used to perform all analyses is provided as an Rmarkdown file (BS_oxBS_Analysis_Kochmanski.Rmd) and HTML file (BS_oxBS_Analysis_Kochmanski.html). Quality control figures from both control probe tests and ChAMP are provided in labeled folders (ChAMP_Raw, ChAMP_ssNoob, CHAMP_SVD_BS, CHAMP_SVD_OX, and Control_Probes). [file Presentation_1.zip › Output copy/Control_Probes/BISULFITE_CONVERSION_II.jpg]

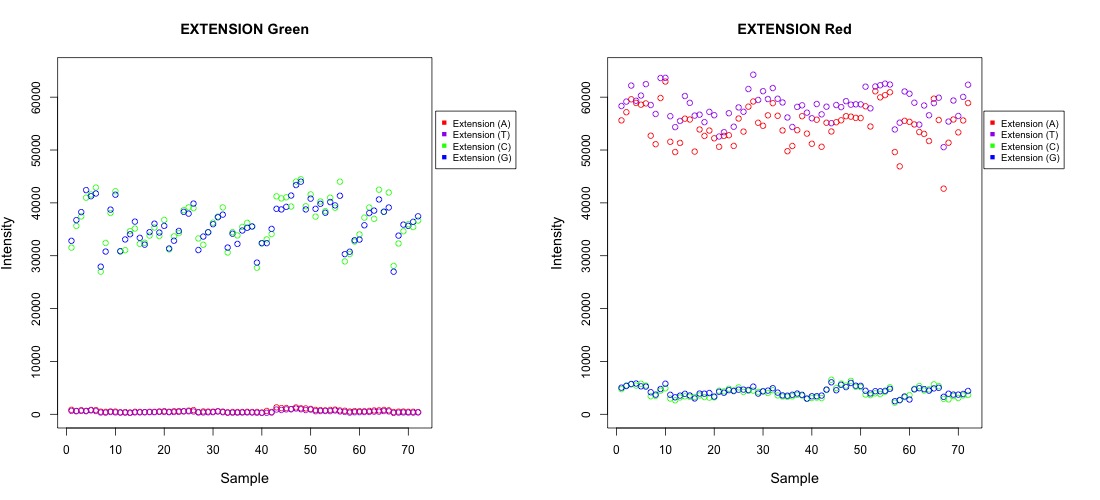

Supplement: Supplementary Material — List of significant differential probes, including those from interaction term modeling (5mC_5hmC_interaction_probes.csv), those from separate models for each epigenetic mark (5mC_probes.csv, 5hmC_probes.csv), and those probes that overlapped between the separate models (5mC_5hmC_overlap_probes.csv). The code used to perform all analyses is provided as an Rmarkdown file (BS_oxBS_Analysis_Kochmanski.Rmd) and HTML file (BS_oxBS_Analysis_Kochmanski.html). Quality control figures from both control probe tests and ChAMP are provided in labeled folders (ChAMP_Raw, ChAMP_ssNoob, CHAMP_SVD_BS, CHAMP_SVD_OX, and Control_Probes). [file Presentation_1.zip › Output copy/Control_Probes/EXTENSION.jpg]

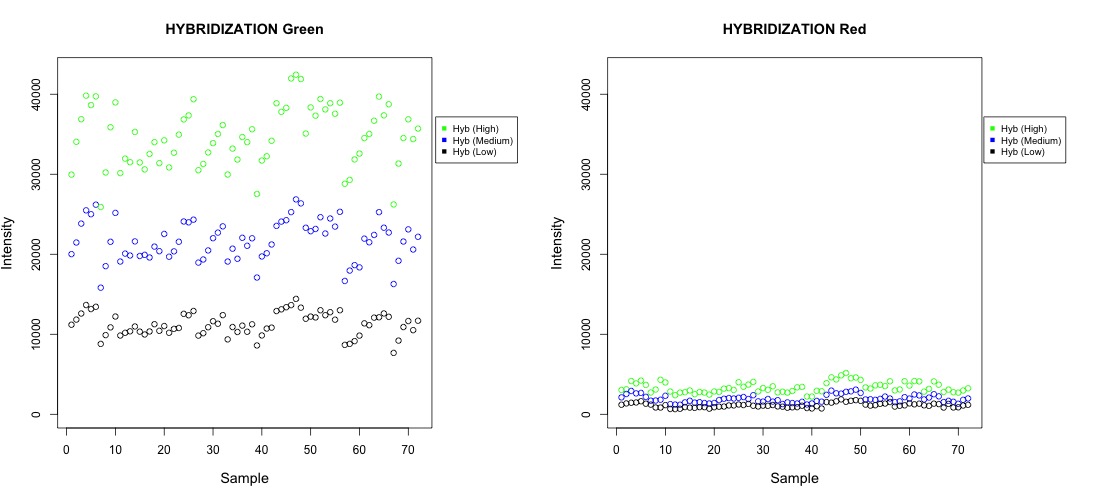

Supplement: Supplementary Material — List of significant differential probes, including those from interaction term modeling (5mC_5hmC_interaction_probes.csv), those from separate models for each epigenetic mark (5mC_probes.csv, 5hmC_probes.csv), and those probes that overlapped between the separate models (5mC_5hmC_overlap_probes.csv). The code used to perform all analyses is provided as an Rmarkdown file (BS_oxBS_Analysis_Kochmanski.Rmd) and HTML file (BS_oxBS_Analysis_Kochmanski.html). Quality control figures from both control probe tests and ChAMP are provided in labeled folders (ChAMP_Raw, ChAMP_ssNoob, CHAMP_SVD_BS, CHAMP_SVD_OX, and Control_Probes). [file Presentation_1.zip › Output copy/Control_Probes/HYBRIDIZATION.jpg]

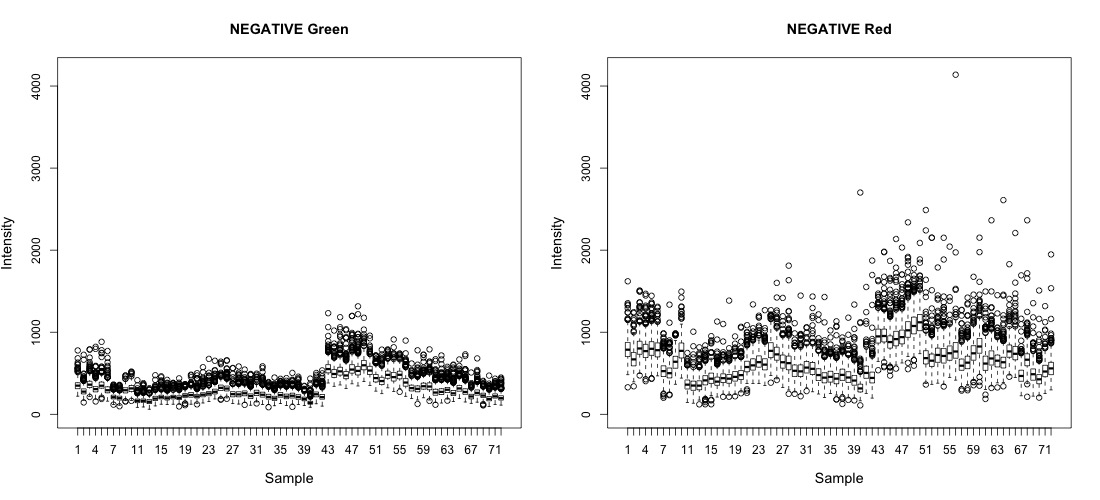

Supplement: Supplementary Material — List of significant differential probes, including those from interaction term modeling (5mC_5hmC_interaction_probes.csv), those from separate models for each epigenetic mark (5mC_probes.csv, 5hmC_probes.csv), and those probes that overlapped between the separate models (5mC_5hmC_overlap_probes.csv). The code used to perform all analyses is provided as an Rmarkdown file (BS_oxBS_Analysis_Kochmanski.Rmd) and HTML file (BS_oxBS_Analysis_Kochmanski.html). Quality control figures from both control probe tests and ChAMP are provided in labeled folders (ChAMP_Raw, ChAMP_ssNoob, CHAMP_SVD_BS, CHAMP_SVD_OX, and Control_Probes). [file Presentation_1.zip › Output copy/Control_Probes/NEGATIVE.jpg]

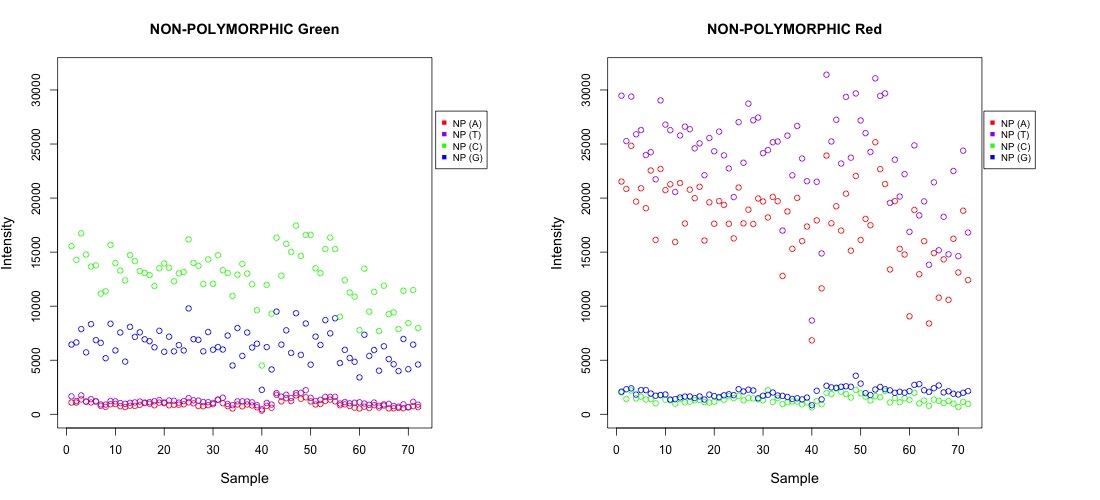

Supplement: Supplementary Material — List of significant differential probes, including those from interaction term modeling (5mC_5hmC_interaction_probes.csv), those from separate models for each epigenetic mark (5mC_probes.csv, 5hmC_probes.csv), and those probes that overlapped between the separate models (5mC_5hmC_overlap_probes.csv). The code used to perform all analyses is provided as an Rmarkdown file (BS_oxBS_Analysis_Kochmanski.Rmd) and HTML file (BS_oxBS_Analysis_Kochmanski.html). Quality control figures from both control probe tests and ChAMP are provided in labeled folders (ChAMP_Raw, ChAMP_ssNoob, CHAMP_SVD_BS, CHAMP_SVD_OX, and Control_Probes). [file Presentation_1.zip › Output copy/Control_Probes/NON-POLYMORPHIC.jpg]

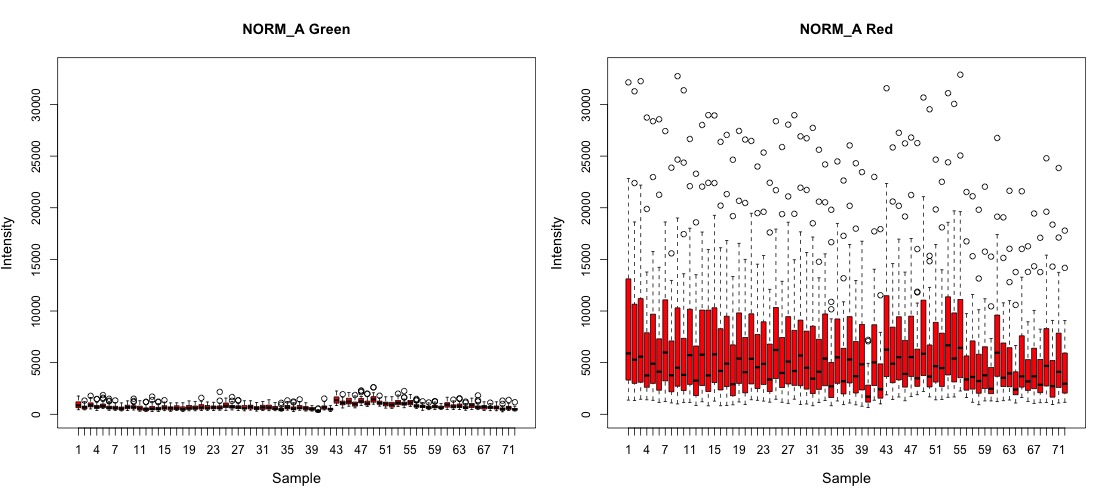

Supplement: Supplementary Material — List of significant differential probes, including those from interaction term modeling (5mC_5hmC_interaction_probes.csv), those from separate models for each epigenetic mark (5mC_probes.csv, 5hmC_probes.csv), and those probes that overlapped between the separate models (5mC_5hmC_overlap_probes.csv). The code used to perform all analyses is provided as an Rmarkdown file (BS_oxBS_Analysis_Kochmanski.Rmd) and HTML file (BS_oxBS_Analysis_Kochmanski.html). Quality control figures from both control probe tests and ChAMP are provided in labeled folders (ChAMP_Raw, ChAMP_ssNoob, CHAMP_SVD_BS, CHAMP_SVD_OX, and Control_Probes). [file Presentation_1.zip › Output copy/Control_Probes/NORM_A.jpg]

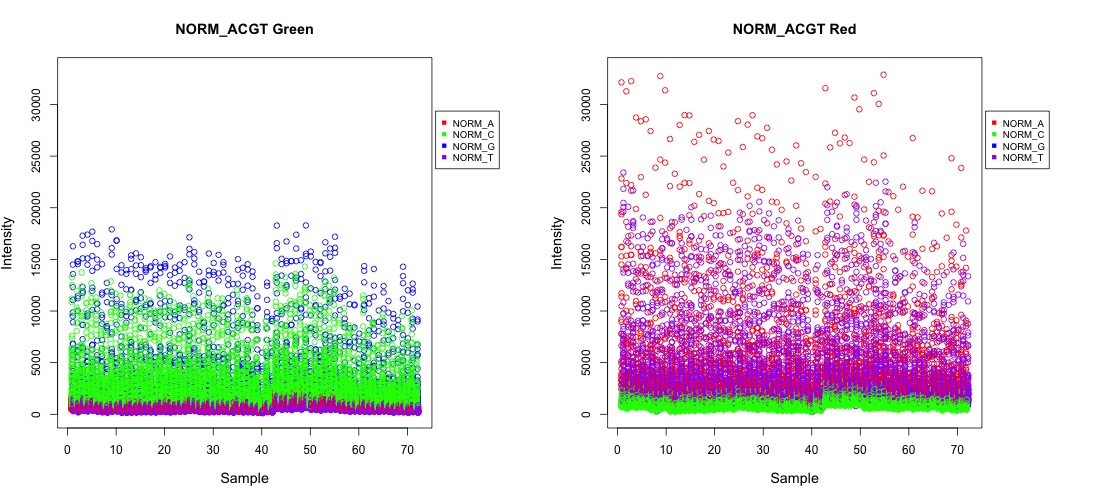

Supplement: Supplementary Material — List of significant differential probes, including those from interaction term modeling (5mC_5hmC_interaction_probes.csv), those from separate models for each epigenetic mark (5mC_probes.csv, 5hmC_probes.csv), and those probes that overlapped between the separate models (5mC_5hmC_overlap_probes.csv). The code used to perform all analyses is provided as an Rmarkdown file (BS_oxBS_Analysis_Kochmanski.Rmd) and HTML file (BS_oxBS_Analysis_Kochmanski.html). Quality control figures from both control probe tests and ChAMP are provided in labeled folders (ChAMP_Raw, ChAMP_ssNoob, CHAMP_SVD_BS, CHAMP_SVD_OX, and Control_Probes). [file Presentation_1.zip › Output copy/Control_Probes/NORM_ACGT.jpg]

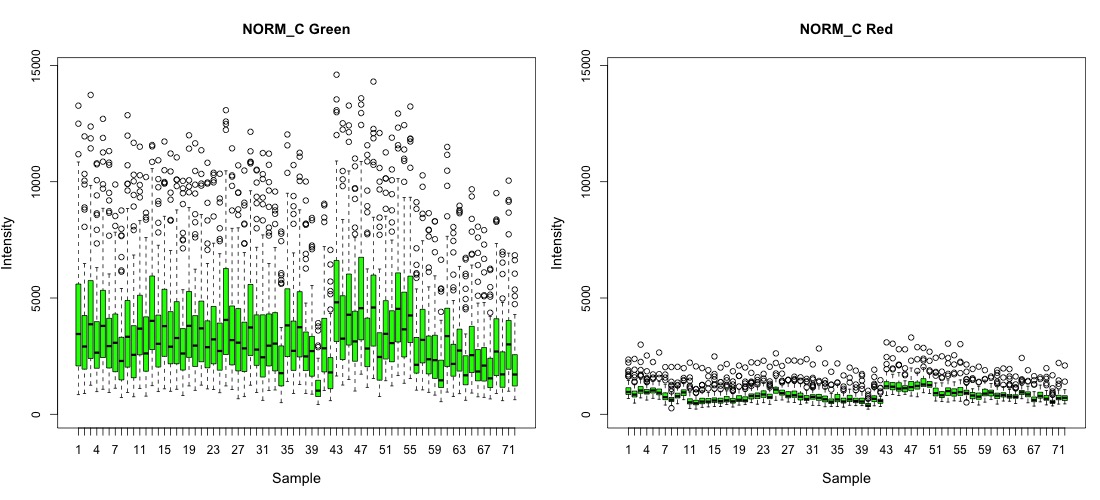

Supplement: Supplementary Material — List of significant differential probes, including those from interaction term modeling (5mC_5hmC_interaction_probes.csv), those from separate models for each epigenetic mark (5mC_probes.csv, 5hmC_probes.csv), and those probes that overlapped between the separate models (5mC_5hmC_overlap_probes.csv). The code used to perform all analyses is provided as an Rmarkdown file (BS_oxBS_Analysis_Kochmanski.Rmd) and HTML file (BS_oxBS_Analysis_Kochmanski.html). Quality control figures from both control probe tests and ChAMP are provided in labeled folders (ChAMP_Raw, ChAMP_ssNoob, CHAMP_SVD_BS, CHAMP_SVD_OX, and Control_Probes). [file Presentation_1.zip › Output copy/Control_Probes/NORM_C.jpg]

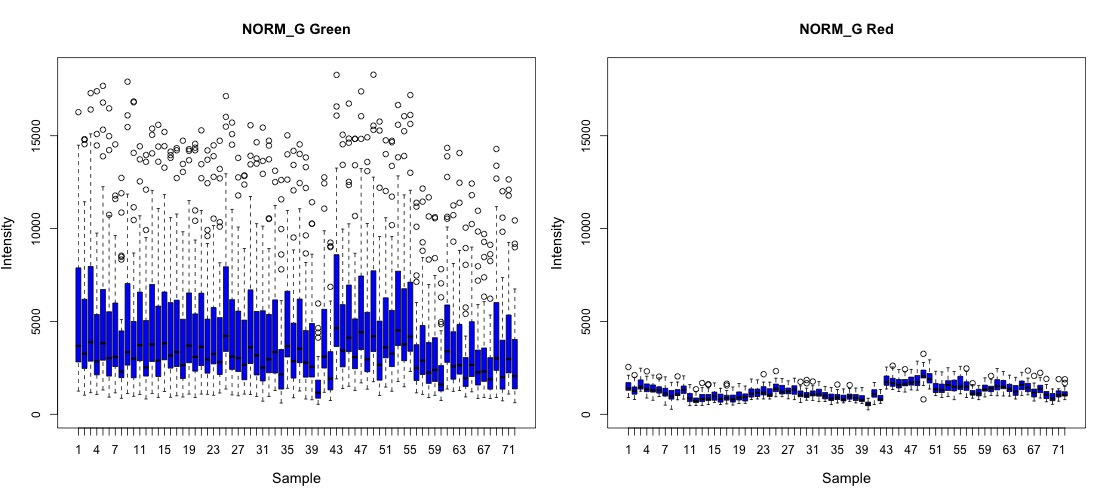

Supplement: Supplementary Material — List of significant differential probes, including those from interaction term modeling (5mC_5hmC_interaction_probes.csv), those from separate models for each epigenetic mark (5mC_probes.csv, 5hmC_probes.csv), and those probes that overlapped between the separate models (5mC_5hmC_overlap_probes.csv). The code used to perform all analyses is provided as an Rmarkdown file (BS_oxBS_Analysis_Kochmanski.Rmd) and HTML file (BS_oxBS_Analysis_Kochmanski.html). Quality control figures from both control probe tests and ChAMP are provided in labeled folders (ChAMP_Raw, ChAMP_ssNoob, CHAMP_SVD_BS, CHAMP_SVD_OX, and Control_Probes). [file Presentation_1.zip › Output copy/Control_Probes/NORM_G.jpg]

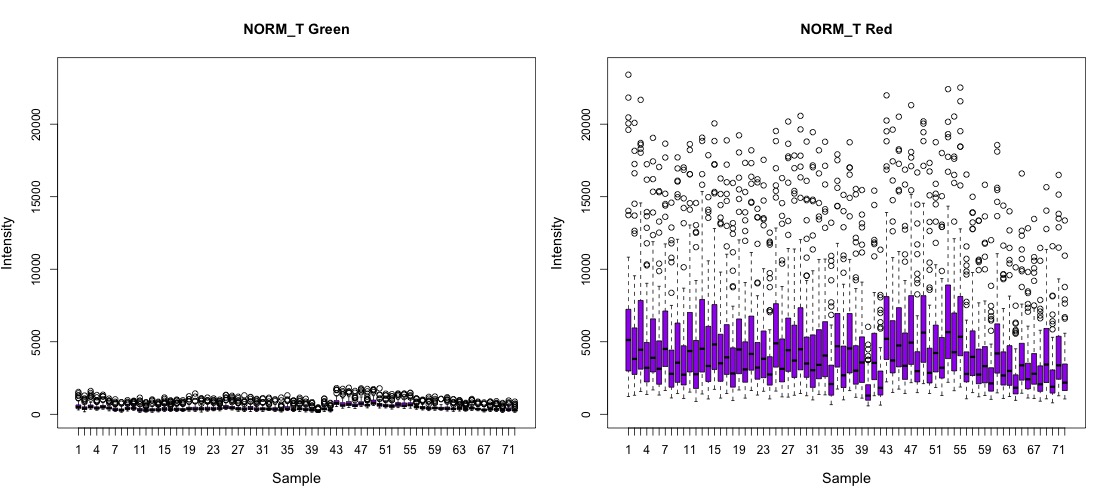

Supplement: Supplementary Material — List of significant differential probes, including those from interaction term modeling (5mC_5hmC_interaction_probes.csv), those from separate models for each epigenetic mark (5mC_probes.csv, 5hmC_probes.csv), and those probes that overlapped between the separate models (5mC_5hmC_overlap_probes.csv). The code used to perform all analyses is provided as an Rmarkdown file (BS_oxBS_Analysis_Kochmanski.Rmd) and HTML file (BS_oxBS_Analysis_Kochmanski.html). Quality control figures from both control probe tests and ChAMP are provided in labeled folders (ChAMP_Raw, ChAMP_ssNoob, CHAMP_SVD_BS, CHAMP_SVD_OX, and Control_Probes). [file Presentation_1.zip › Output copy/Control_Probes/NORM_T.jpg]

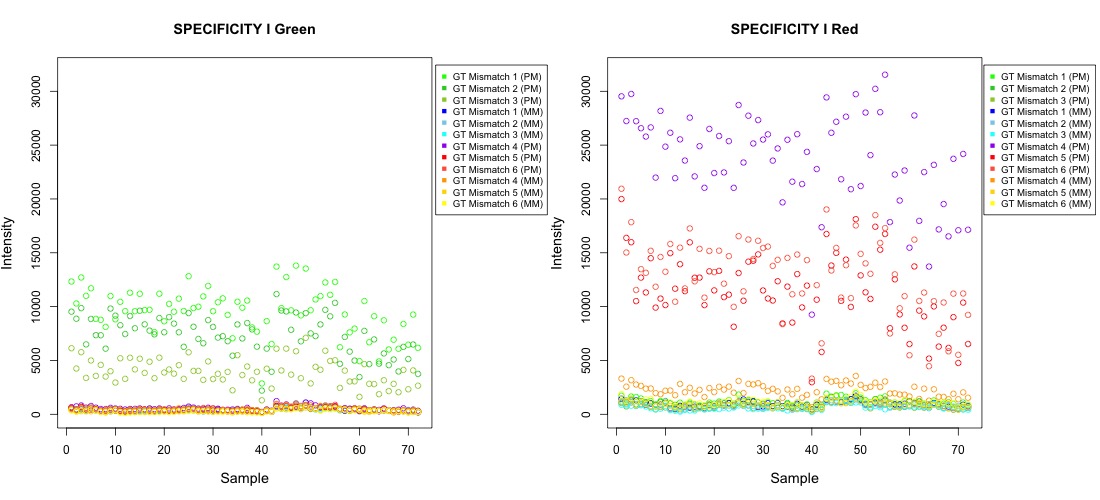

Supplement: Supplementary Material — List of significant differential probes, including those from interaction term modeling (5mC_5hmC_interaction_probes.csv), those from separate models for each epigenetic mark (5mC_probes.csv, 5hmC_probes.csv), and those probes that overlapped between the separate models (5mC_5hmC_overlap_probes.csv). The code used to perform all analyses is provided as an Rmarkdown file (BS_oxBS_Analysis_Kochmanski.Rmd) and HTML file (BS_oxBS_Analysis_Kochmanski.html). Quality control figures from both control probe tests and ChAMP are provided in labeled folders (ChAMP_Raw, ChAMP_ssNoob, CHAMP_SVD_BS, CHAMP_SVD_OX, and Control_Probes). [file Presentation_1.zip › Output copy/Control_Probes/SPECIFICITY_I.jpg]

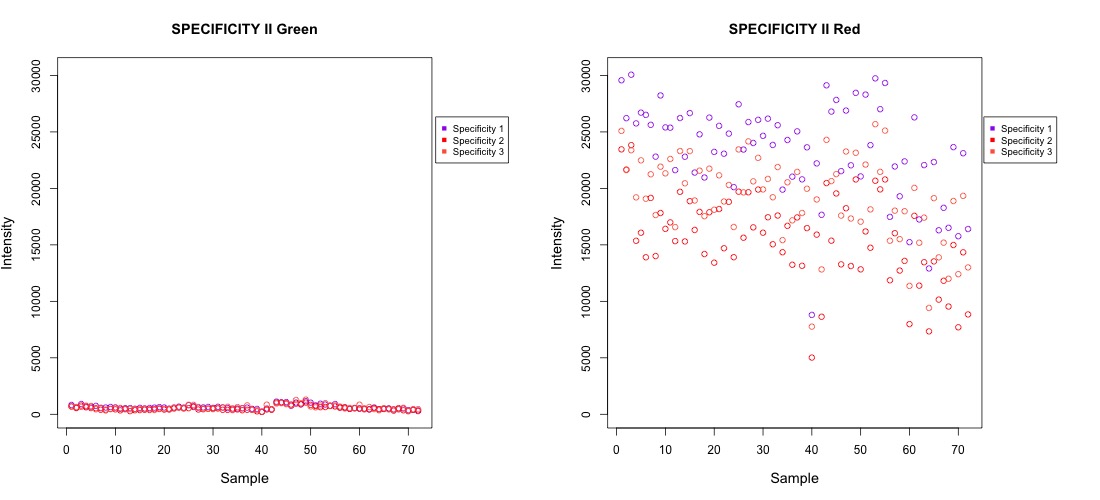

Supplement: Supplementary Material — List of significant differential probes, including those from interaction term modeling (5mC_5hmC_interaction_probes.csv), those from separate models for each epigenetic mark (5mC_probes.csv, 5hmC_probes.csv), and those probes that overlapped between the separate models (5mC_5hmC_overlap_probes.csv). The code used to perform all analyses is provided as an Rmarkdown file (BS_oxBS_Analysis_Kochmanski.Rmd) and HTML file (BS_oxBS_Analysis_Kochmanski.html). Quality control figures from both control probe tests and ChAMP are provided in labeled folders (ChAMP_Raw, ChAMP_ssNoob, CHAMP_SVD_BS, CHAMP_SVD_OX, and Control_Probes). [file Presentation_1.zip › Output copy/Control_Probes/SPECIFICITY_II.jpg]

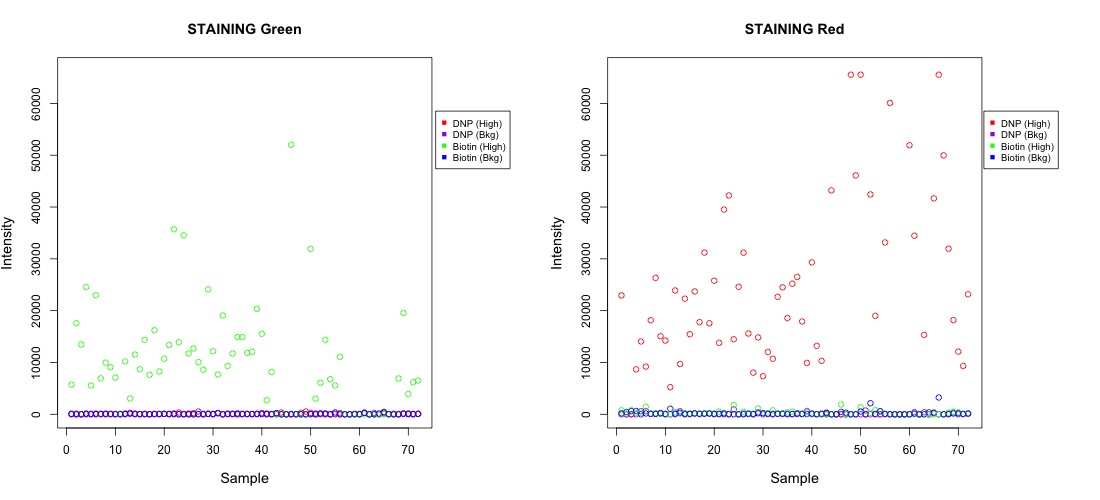

Supplement: Supplementary Material — List of significant differential probes, including those from interaction term modeling (5mC_5hmC_interaction_probes.csv), those from separate models for each epigenetic mark (5mC_probes.csv, 5hmC_probes.csv), and those probes that overlapped between the separate models (5mC_5hmC_overlap_probes.csv). The code used to perform all analyses is provided as an Rmarkdown file (BS_oxBS_Analysis_Kochmanski.Rmd) and HTML file (BS_oxBS_Analysis_Kochmanski.html). Quality control figures from both control probe tests and ChAMP are provided in labeled folders (ChAMP_Raw, ChAMP_ssNoob, CHAMP_SVD_BS, CHAMP_SVD_OX, and Control_Probes). [file Presentation_1.zip › Output copy/Control_Probes/STAINING.jpg]

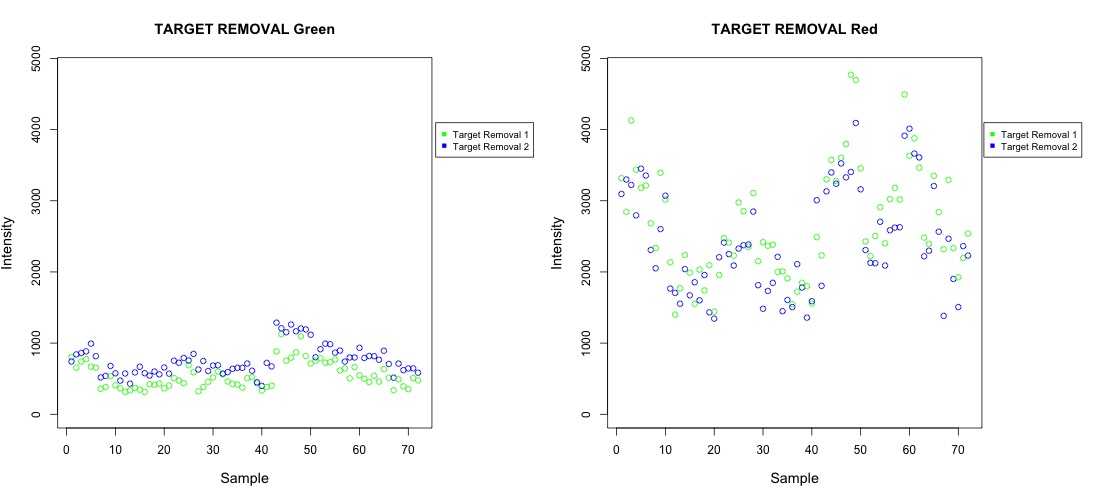

Supplement: Supplementary Material — List of significant differential probes, including those from interaction term modeling (5mC_5hmC_interaction_probes.csv), those from separate models for each epigenetic mark (5mC_probes.csv, 5hmC_probes.csv), and those probes that overlapped between the separate models (5mC_5hmC_overlap_probes.csv). The code used to perform all analyses is provided as an Rmarkdown file (BS_oxBS_Analysis_Kochmanski.Rmd) and HTML file (BS_oxBS_Analysis_Kochmanski.html). Quality control figures from both control probe tests and ChAMP are provided in labeled folders (ChAMP_Raw, ChAMP_ssNoob, CHAMP_SVD_BS, CHAMP_SVD_OX, and Control_Probes). [file Presentation_1.zip › Output copy/Control_Probes/TARGET_REMOVAL.jpg]
