# Supplementary material for: A Novel Application of Mixed Effects Models for Reconciling Base-Pair Resolution 5-Methylcytosine and 5-Hydroxymethylcytosine Data in Neuroepigenetics
Source: Front Genet. 2019 Sep 10;10:801. doi: 10.3389/fgene.2019.00801 (PMC6748167; doi:10.3389/fgene.2019.00801)
Supplement: Supplementary Material — List of significant differential probes, including those from interaction term modeling (5mC_5hmC_interaction_probes.csv), those from separate models for each epigenetic mark (5mC_probes.csv, 5hmC_probes.csv), and those probes that overlapped between the separate models (5mC_5hmC_overlap_probes.csv). The code used to perform all analyses is provided as an Rmarkdown file (BS_oxBS_Analysis_Kochmanski.Rmd) and HTML file (BS_oxBS_Analysis_Kochmanski.html). Quality control figures from both control probe tests and ChAMP are provided in labeled folders (ChAMP_Raw, ChAMP_ssNoob, CHAMP_SVD_BS, CHAMP_SVD_OX, and Control_Probes). [file raw_mdsPlot.pdf › Output copy/ChAMP_ssNoob/raw_mdsPlot.pdf]

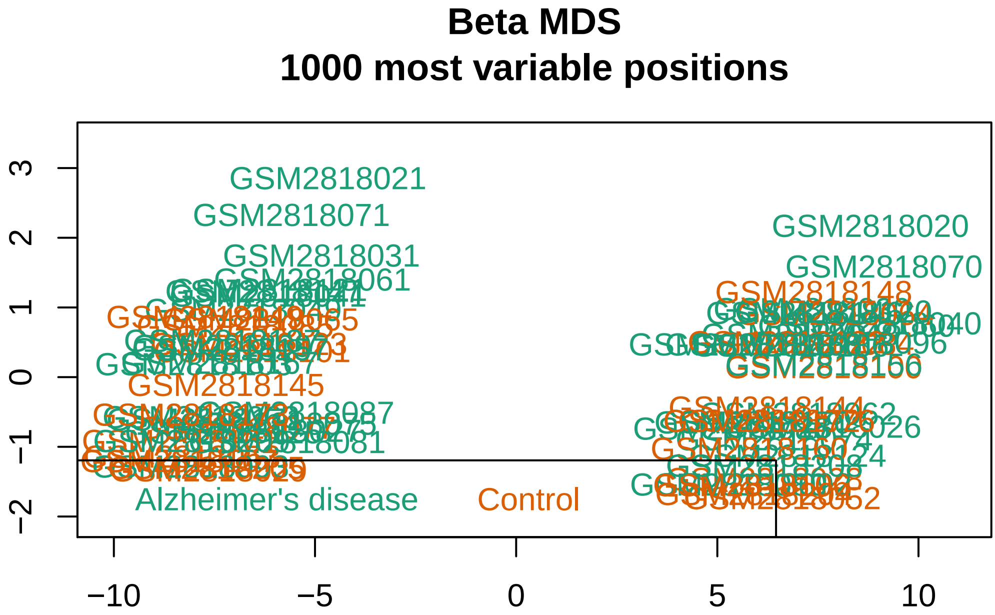
Beta MDS
1000 most variable positions
GSM2818021
GSM2818071
GSM2818031
GSM2818061
GSM2818020
GSM2818070
GSM2818148
GSM2818145
GSM2818087
GSM2818081
GSM2818144
GSM2818156
GSM2818052
Alzheimer's disease
Control
3
2
1
0
−1
−2
−10
−5
0
5
10
